# Identification of molecular subtypes and a prognostic signature based on chromatin regulators related genes in prostate cancer

**DOI:** 10.3389/fgene.2022.1110723

**Published:** 2023-01-10

**Authors:** Hangbin Ma, Cheng Zhou, Jianchao Ge, Wandong Yu, Yinghao Zhou, Pengyu Wang, Xuehu Zhang, Jun Zhang, Guowei Shi

**Affiliations:** Department of Urology, Shanghai Fifth People’s Hospital, Fudan University, Shanghai, China

**Keywords:** prostate cancer, chromatin regulators, MXD3, prognostic model, molecular subtypes

## Abstract

The clinical and molecular phenotypes of prostate cancer (PCa) exhibit substantial heterogeneity, ranging from indolent to metastatic disease. In this study, we aimed to identify PCa subtypes and construct a gene signature that can predict the recurrence-free survival (RFS) of PCa patients based on chromatin regulators genes (CRGs). Strikingly, we identified two heterogeneous subtypes with distinct clinical and molecular characteristics. Furthermore, by performing differential analysis between the two CRGs subtypes, we successfully constructed a gene signature to predict PCa prognosis. The signature, comprising four genes (MXD3, SSTR1, AMH and PPFIA2), was utilized to classify PCa patients into two risk groups; the high-risk group was characterized by poor prognosis and more aggressive clinical features. Moreover, we investigated the immune profile, mutation landscape and molecular pathways in each of the groups. Additionally, drug-susceptibility testing was performed to explore sensitive drugs for high-risk patients. Furthermore, we found that MXD3 downregulation suppressed the proliferation of PCa cell lines *in vitro*. Overall, our results highlight the signature based on CRGs as a powerful tool for predicting RFS of PCa patients, as well as an indicator for personalized treatment of those patients.

## Introduction

Prostate cancer (PCa) is among the leading causes of cancer-related death in men, with approximately 350,000 deaths annually (Sung et al., 2021). Patients with localized disease at a low to intermediate risk of recurrence generally have a favorable outcome of 99% 10-year overall survival if the disease is detected and treated at an early stage (Rebello et al., 2021). However, patients with metastatic androgen-independent prostate cancer (mCRPC) have progressive and morbid disease with a median survival of 10–12 months (Attard et al., 2016). Therefore, considering the substantial prognostic differences, there is a need to develop biomarkers with the potential to improve prognostic classification of PCa. The pathogenesis, tumor outcomes, and pathological types of PCa are strongly correlated with gene mutations and epigenetic changes, along with factors such as age, ethnicity and family history (Sinha et al., 2019; Ge et al., 2020; Li et al., 2020). Importantly, nearly half of the interindividual variation in PCa risk can be attributed to genetic factors (Mucci et al., 2016). For instance, men with a germline mutation in BRCA2 or HOXB13 have an approximately eightfold to ninefold higher and approximately threefold higher risk, respectively, of developing PCa than men without a mutation (Kote-Jarai et al., 2011; Karlsson et al., 2014; Kote-Jarai et al., 2015; Merseburger et al., 2021). Additionally, MYC is almost ubiquitously expressed at each stage of tumor development; it can be upregulated through direct transcriptional targeting by many other genes, thereby driving proliferation and therapeutic resistance (Hubbard et al., 2016). Furthermore, epigenetic alterations play important roles in promoting PCa. One study showed that 22% of mCRPC patients exhibited a novel epigenomic subtype associated with hypermethylation and somatic mutations in TET2, DNMT3B, IDH1, and BRAF (Zhao et al., 2020). Clinical and preclinical studies have revealed epigenetic alterations (DNA methylation, histone modification, and chromatin remodeling) that may be useful in distinguishing among aggressive types of prostate tumors (Kumaraswamy et al., 2021).

Chromatin regulator (CRs) are clusters of regulatory elements with distinct functions that are strongly related to genetic changes and epigenetic alterations (Keung et al., 2014; Lu et al., 2018). Chromatin regulators are indispensable upstream regulatory factors of epigenetics that modify chromatin in unique combinatorial, spatial, and temporal patterns (Keung et al., 2014). Altered epigenetic regulation of genomic activity is important in tumorigenesis, and multiple CRs exhibit dysregulated gene expression patterns across cancer types. Furthermore, high-resolution genome-sequencing efforts have discovered numerous mutations in genes encoding epigenetic regulators that have roles as “writers”, “readers”, ‘“editors” of CRs which act as DNA methylator and/or chromatin states (Plass et al., 2013; Lu et al., 2018). Lei Gu et al. found that overexpression of the gene encoding BAZ2A (TIP5) is involved in PCa-related epigenetic alterations that lead to disease recurrence (Gu et al., 2015). Mutations in epigenetic pathways highlight the importance of links between gene defects and epigenetic changes. Although there remains limited integration of cancer epigenetic profiles with cancer genetic profiles, a CR-based signature may be useful in forecasting the clinical prognosis of PCa patients.

In this study, we screened chromatin regulator (CRs) related to PCa prognosis through differential analysis and Cox analysis. On the basis of consistent clustering findings, we identified stable molecular subtypes with different prognostic and pathological characteristics. Furthermore, we used differential analysis to develop a prognostic signature based on CRs subtypes. Our findings highlight recurrence-free survival (RFS) stratification, somatic mutations, immune cell infiltration, nomogram construction and potential drug prediction according to risk characteristics. Finally, we selected the hub gene MXD3 for further validation *in vitro*.

## Materials and methods

### Patient gene profile and clinical information data collection

This study enrolled two independent PCa cohorts. Firstly, Gene expression (RNA-seq) transcriptome (raw counts and transcripts per million reads (TPM)) and sample clinical profiles were downloaded from The Cancer Genome Atlas (TCGA) database (2022.5.1) (https://portal.gdc.cancer.gov/). Certain clinical characteristics, such as Gleason score, which were not accessible in the Genomic Data Commons (GDC) data portal were curated from UCSC XENA (https://xenabrowser.net/datapages/). In total, 491 patients were incorporated into this study. GSE70770 cohort, including 203 PCa patients with microarray expression profile and matched clinicopathological information were curated from the Gene Expression Omnibus (GEO) database (https://www.ncbi.nlm.nih.gov/gds/). The baseline characteristics of patients in the two cohorts are shown in Supplementary Table S1. Chromatin regulators (CRs) were summarized from the previous literature (Lu et al., 2018).

### Differential expression analysis and functional enrichment analysis

We performed DESeq2 algorithm using DESeq2 R package (R v1.36.0) to explore differentially expressed genes between the normal prostate and tumor part. LfcShrink and “ashr” method was used to generate more accurate estimates (Love et al., 2014). Differential expressed chromatin regulator genes (DE-CRGs) were a

cquired by comparing normal and tumor part in TCGA and filter criteria was |Log2Foldchange| greater than 1.2 and FDR lower than 0.05. “ClusterProfiler” R package (R v4.4.4) was applied to perform Gene Oncology (GO) functional enrichment analysis (Yu et al., 2012). Furthermore, we visualized co-expression potential using the R package corrplot (R v4.1.2), to easily present co-expression and anti-correlation between genes.

## Chromatin regulators genes-based clustering

We firstly identified DE-CRGs associated with recurrence-free survival (RFS) by performing univariate Cox regression analysis (*p* < 0.05). And the heatmap was further used to present the correlation between these genes, which was assessed by chi-square test. Then “ConsensusClusterPlus” R package (R v1.54.0) was utilized to perform cluster analysis to identify CRs-related subtypes (Wilkerson and Hayes, 2010). For verification of the distinct classification between Cluster 1 and Cluster2, we conducted principal component analysis (PCA) using “prcomp” function in R (v4.0.4). We performed 1,000 times repetitions to determine the stability of this classification. Kaplan-Meier (K-M) analysis was further used to compare the clinical outcome between the two clusters.

### Clinical and immune scores specific for the CRs-related subtype

Besides, chi-square test was also utilized to assess clinicopathological characteristics distribution between two CRs-related subtypes. Next, we obtained ten oncogenic pathways which are somatically altered in varying cancers. And ssGSEA algorithm was performed to explore the distinct of the ten oncogenic pathways between two CRs-related subtypes (Sanchez-Vega et al., 2018). Moreover, the somatic mutation transcriptome of PCa patients was downloaded from the TCGA database. We analyzed the somatic mutation data by using the “maftools” R package (Mayakonda et al., 2018). We further applied a metagene approach utilized previously for immune cell subpopulations for PRAD tumor microenvironment evaluation (Bindea et al., 2013). The gene set variation analysis (GSVA) method was used to estimate the relative infiltration score of immune cells (Hänzelmann et al., 2013). Furthermore, immune profile differences between two subgroups were assessed by Wilcoxon test.

### Signature development and validation based on CRs-related subtype

Firstly, the differentially expressed genes (DEGs) between two subtypes were obtained by using the R package “limma” (R v3.52.2) (Foldchange> 1.5 and FDR <0.05) (Ritchie et al., 2015). Then we intersected these DEGs with differentially expressed genes between the normal prostate and tumor part in TCGA database for subsequent analysis. We then used univariate Cox regression analysis to identify genes correlated with RFS in the training cohort (*p* < 0.05). The least absolute shrinkage and selection operator (LASSO) algorithm was applied to remove the overfitting between the prognosis-associated genes and reduce the scope of the prognosis-associated genes with penalty parameter tuning conducted *via* 10−fold cross−validation according to the R package “glmnet”. Next, the genes curated from LASSO regression analysis were incorporated in the multivariate Cox regression analysis. The signature risk score was calculated according to the average expression of each gene and matched regression coefficients generated from multivariate Cox regression analysis. The risk score formula was calculated as follows:

Risk score = betagene1× exprgene1+ betagene2×exprgene2+ betagenen × exprgene.

Then the patients were clustered into high- and low-risk groups based on the median risk score. In addition to the Kaplan–Meier survival curves, we also applied the time-dependent receiver operational feature curves (ROC) generated by the R packages “suvminer” and “survival ROC” to evaluate the performance of the signature risk score in predicting the RFS of PCa patient.

### Construction of a nomogram including risk score and clinical characteristics

Independent prognostic factors and related clinical parameters were obtained from Cox stepwise regression analysis for constructing a prognostic nomogram to predict 1-, 2-, and 3-year RFS for PCa patients. Then we used Calibration curves of 1-, 2-, and 3-year to evaluate the reliability of this nomogram. Moreover, we also utilized the decision curve analysis (DCA) to compare the performance of clinical parameters and the nomogram model.

### Comparison of clinical parameters, immune scores and TMB between different risk group

We exerted chi-square tests on the related clinical characteristics in different risk groups including T stage, Gleason score, and clinical outcome. CIBERSORT is an online tool for quantifying the infiltration abundance of 22 types of immune cells according to the basement of linear support vector regression (Chen et al., 2018). Aiming to estimate the immune cell function between the different risk groups, we used CIBERSORT to calculate the abundance of Tumor-infiltrating immune cells (TIICs) in PRAD. We further drew a comparison in the abundance of tumor infiltrating immune cells between the high- and low-risk groups by Wilcoxon test. Besides, the ESTIMATE algorithm was utilized to assess immune infiltration in PRAD patients (Li et al., 2017). Moreover, a Spearman correlation analysis between immune infiltrating cells and risk score or core genes constituting the risk score was performed. Additionally, we also performed chi-square test analysis to explore the TMB differences in the HR and LR subtype.

### Distinct molecular characteristics between high-risk group and low-risk group

To explore the differences of molecular characteristics between the CRGs subgroups, we performed the gene set enrichment analysis (GSEA) based on the “ClusterProfiler” R package. GSVA enrichment analysis was also carried out to compare the transcriptomic remodeling using GSVA R package (R v1.44.2). “Limma” package was also utilized to nominate the distinct molecular pathways between the two subgroups (*p* < 0.05 was considered significant). Besides, we used “pRRophetic” R package to explore the therapeutic sensitivity and the concentration inducing 50% reduction growth (IC50) of targeted inhibitors. Then we performed Wilcoxon test to compare the IC50 difference between the two subgroups.

### Cell culture

The human Pca cell lines PC3 and C4-2 were purchased from the American Type Culture Collection (Manassas, United States) and cultured in RPMI-1640 medium (Corning, Inc., Corning, NY, United States) containing 10% fetal bovine serum (GIBCO) and 1% penicillin/streptomycin (GIBCO). All cells were grown at 37 °C in a 5% CO2 humidified incubator.

### Plasmids and lentivirus infection

Short hairpin RNA (shRNA) expression sequences are documented in Additional file: Table S3. Then these sequences were cloned into the pLKO.1 vector. And plasmids were transfected into HEK293FT cells utilizing PEI 25K (23966–1; Polysciences, Warrington, PA, United States) based on the manufacturer’s instructions. PC3 and C4-2 cells were transduced with lentivirus, and stable transformants were selected with puromycin (5 μg/ml) for 7 days.

### Real-time PCR analysis

RNA was isolated using TRIzol reagent (Invitrogen) in accordance with the manufacturer’s instructions. Then 1 ug of total RNA was reverse transcribed into cDNA using a PrimeScript™ first Strand cDNA Synthesis Kit (6110A; TaKaRa). qRT-PCR was conducted using TB Green Premix ExTaq (Tli RNaseH Plus) (RR420; TaKaRa) on the ABI7500 System (Applied Bio Systems, Foster City, CA, United States). Then we used 2 − ΔΔCt method to calculate the relative expression levels of genes. GAPDH was considered as an internal control for RT-PCR. Primers used to amplify genes of interest were listed in Additional file Table S3.

### Cell growth and colony formation assay

Cell growth was detected by Cell Counting Kit-8 (CK04; Dojindo, Kumamoto, Japan) at indicated time points according to the manufacturer’s instructions. The cells were cultured in 96-well plates (1000 cells per well) for 6 days. Ten μL CCK8 reagent was added to 100 μl complete medium in each well and then cultured at 37 °C for 3 h. Then the absorbance values were assessed using a microplate reader (Tecan, Mechelen, Belgium) at 450 nm (A450). Colony formation assay was conducted by seeding 1000 cells in complete medium for 10–12 days depending on colony size. Then the cells were fixed using methanol for 10 min and stained using 0.5% crystal violet for 1 h. Images were captured following wash of PBS.

### Statistical analysis

Statistical analysis in this study was performed using R software v4.0.4 and Prism software, version 8 (GraphPad Software, San Diego, CA, United States). *p* values less than 0.05 were thought to be statistically significant.

## Results

### Identification of differentially expressed CRs-Related genes and biological function analysis

**FIGURE 1:**
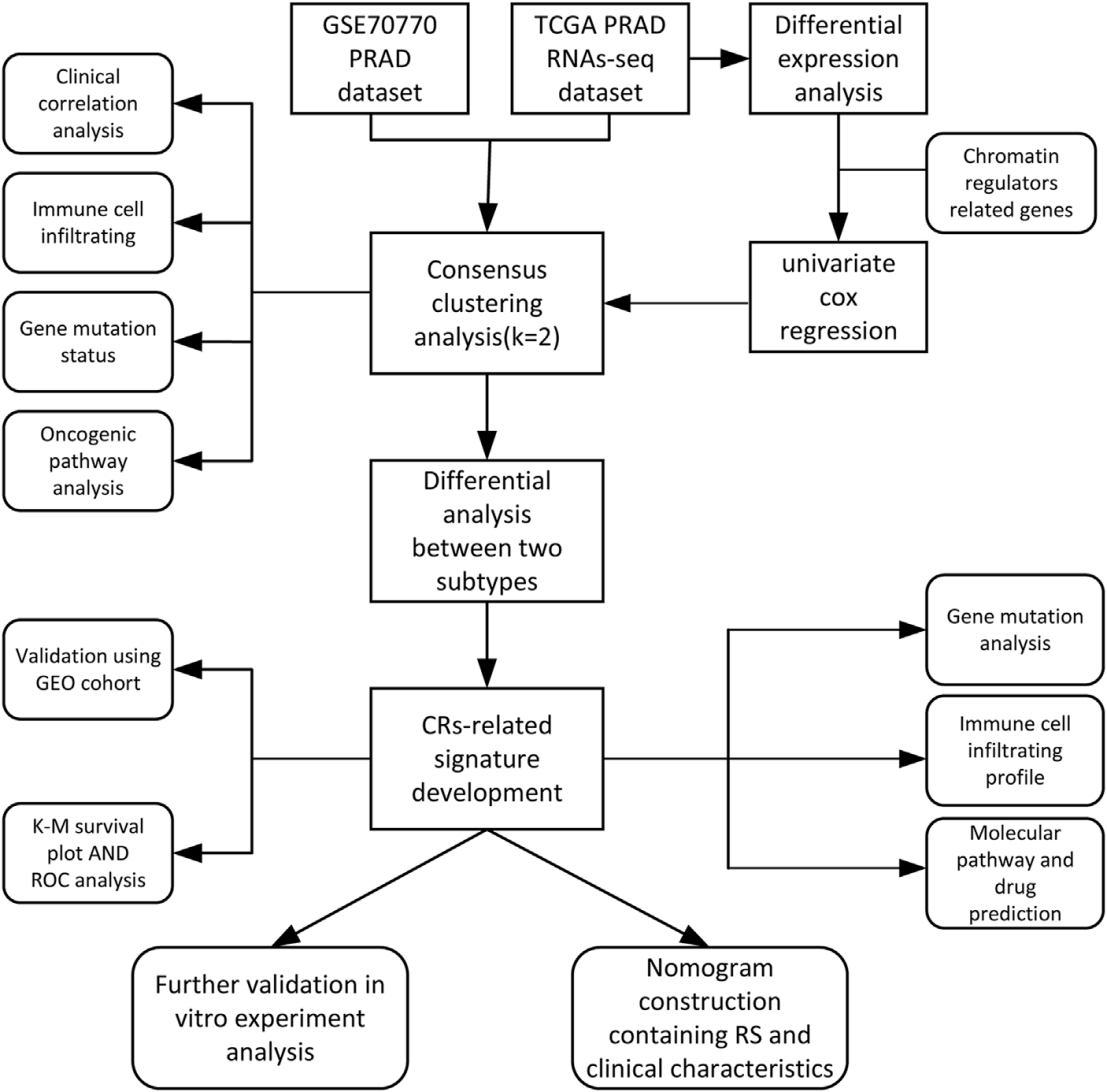
The main analysis workflow.

**FIGURE 2:**
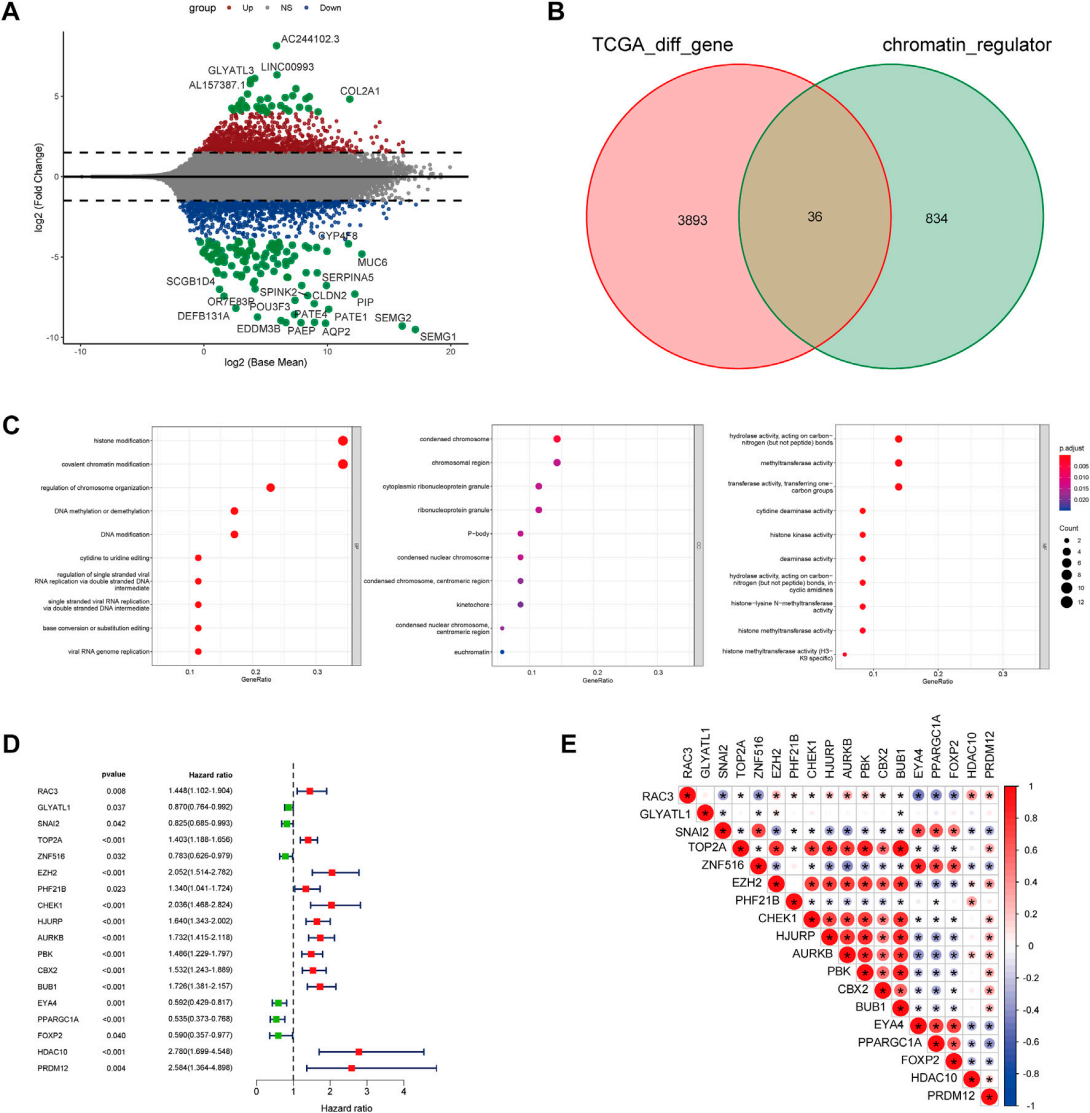
**(A)** Volcano plot showing 2001 up-regulated and 1928 down-regulated genes in TCGA database (*p* < 0.05 and |log2FC| > 1.2) **(B)** Venn diagram used to identify the common CRGs and differentially expressed gene between normal prostate and tumor parts **(C)** The enriched pathways belonged to BP, CC, and MF in GO analysis for DE-CRGs **(D)** Forest plots presents there are 18 prognostics differentially expressed CRGs based on the univariate cox regression analysis. **(E)** The correlations between the prognostic differentially expressed CRGs.

The main analysis workflow is presented in Figure 1. Firstly, we identified differentially expressed genes (DEGs), including 2001 upregulated genes and 1928 downregulated genes by performing differential analysis between prostate tumor and normal part acquired from The Cancer Genome Atlas (TCGA) dataset (Figure 2A). Subsequently, we obtained 36 differentially expressed CR-related genes based on the intersection of CR-related genes and DEGs in TCGA (Figure 2B). Gene Ontology enrichment analysis revealed that the above differentially expressed CR-related genes were mainly enriched in “histone modification” in the biological process category, “condensed chromosome” in the cellular component category and “hydrolase activity, acting on carbon nitrogen (but not peptide) bonds” in the molecular function (Figure 2C). Univariate Cox analysis suggested that 18 CR-related genes are significantly associated with recurrence-free survival (RFS) (Figure 2D). Furthermore, correlation analysis suggested that most genes were significantly associated with each other (Figure 2E).

### Chromatin regulators genes-based clustering

**FIGURE 3:**
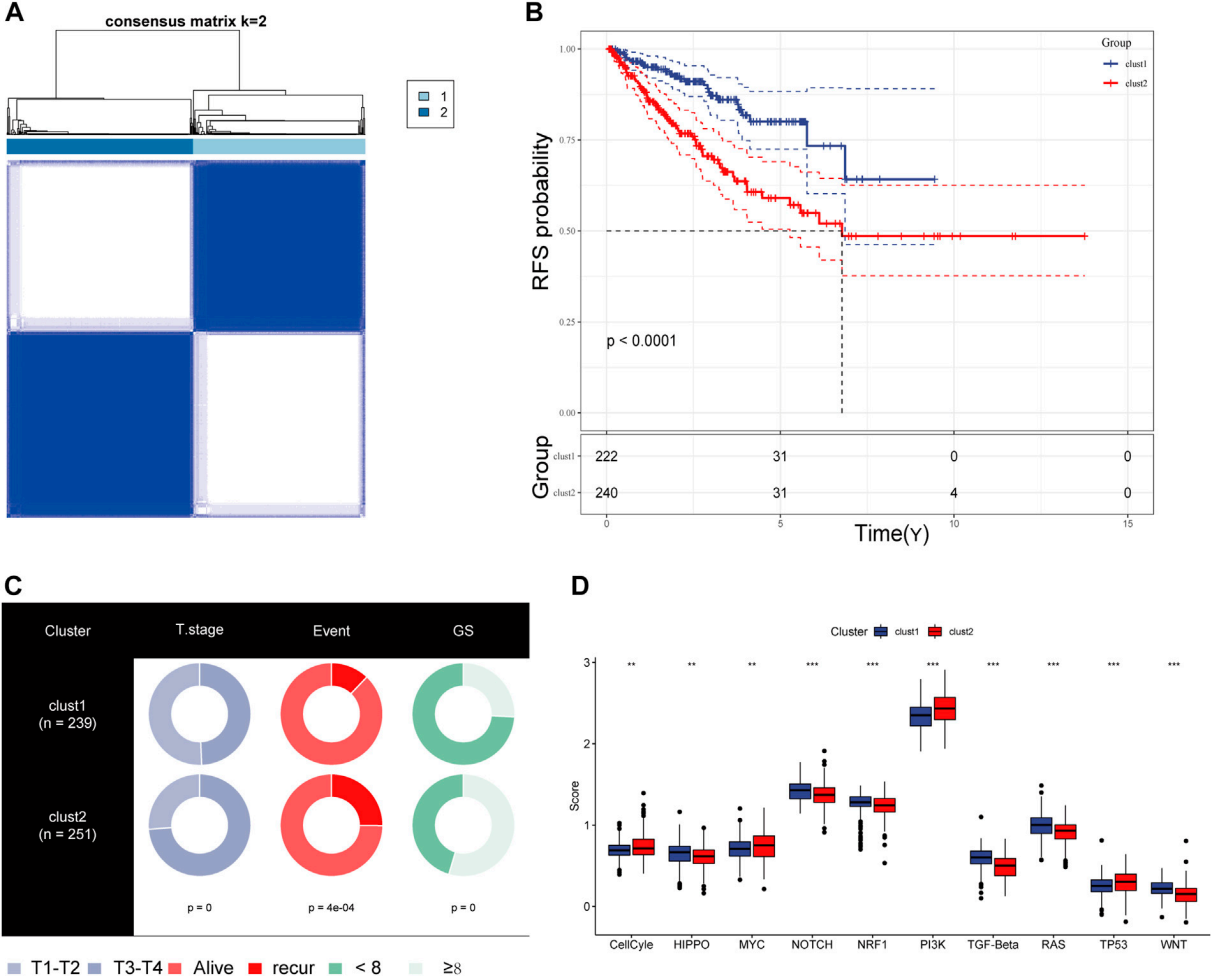
**(A)** The consensus matrix reveals patients with two distinct CRGs subtype in the TCGA dataset. **(B)** Kaplan-Meier curves for recurrence-free survival according to CRGs subtype (Log-rank test) in TCGA dataset. **(C)** Stratified proportion of clinical features of PCa patients in CRGs-related subtype in the TCGA database. **(D)** the distinct of cancer-related pathway in CRGs-related subtype.

For exploration of CRs-related genes heterogeneity in PCa, we applied consensus clustering analysis to construct CRs-related molecular clusters of Pca using a TCGA cohort. The results of the consensus package in R were used to identify two CRs-related subtypes (Figure 3A) (Wilkerson and Hayes, 2010). We conducted principal component analysis (PCA) using “prcomp” function in R (v4.0.4) to verify the distinct classification between Cluster 1 and Cluster2 (Supplementary Figure S1). The two subtypes showed distinct clinical outcomes. Kaplan–Meier (K-M) plots revealed that patients in cluster 2 exhibited inferior RFS (Figure 3B). Similarly, the GSE70770 cohort could also be divided into two subgroups based on the CRs-related genes expression (Supplementary Figure S2A), and K-M plots also showed a similar difference in RFS between the two subtypes (Supplementary Figure S2B). Additionally, comparison of the two subtypes based on clinical parameters revealed that cluster 2 contained a greater proportion of patients with a higher Gleason Score (GS), advanced T stage and recurrent status present more proportions in cluster 2 (Figure 3C). Next, we performed single-sample gene set enrichment analysis (ssGESA) method to compare ten distinct oncogenic pathways between the two subtypes. Notably, the score of oncogenic pathways involved in the deterioration of prostate cancer, including Cell Cycle, MYC and PI3K-AKT pathways, were significantly elevated in cluster2 (Figure 3D). Collectively, these results implied that the clinical and molecular characteristics of PCa were more aggressive among patients in cluster 2.

### Mutational landscape and immune profile specific to CRs-related subtype

**FIGURE 4:**
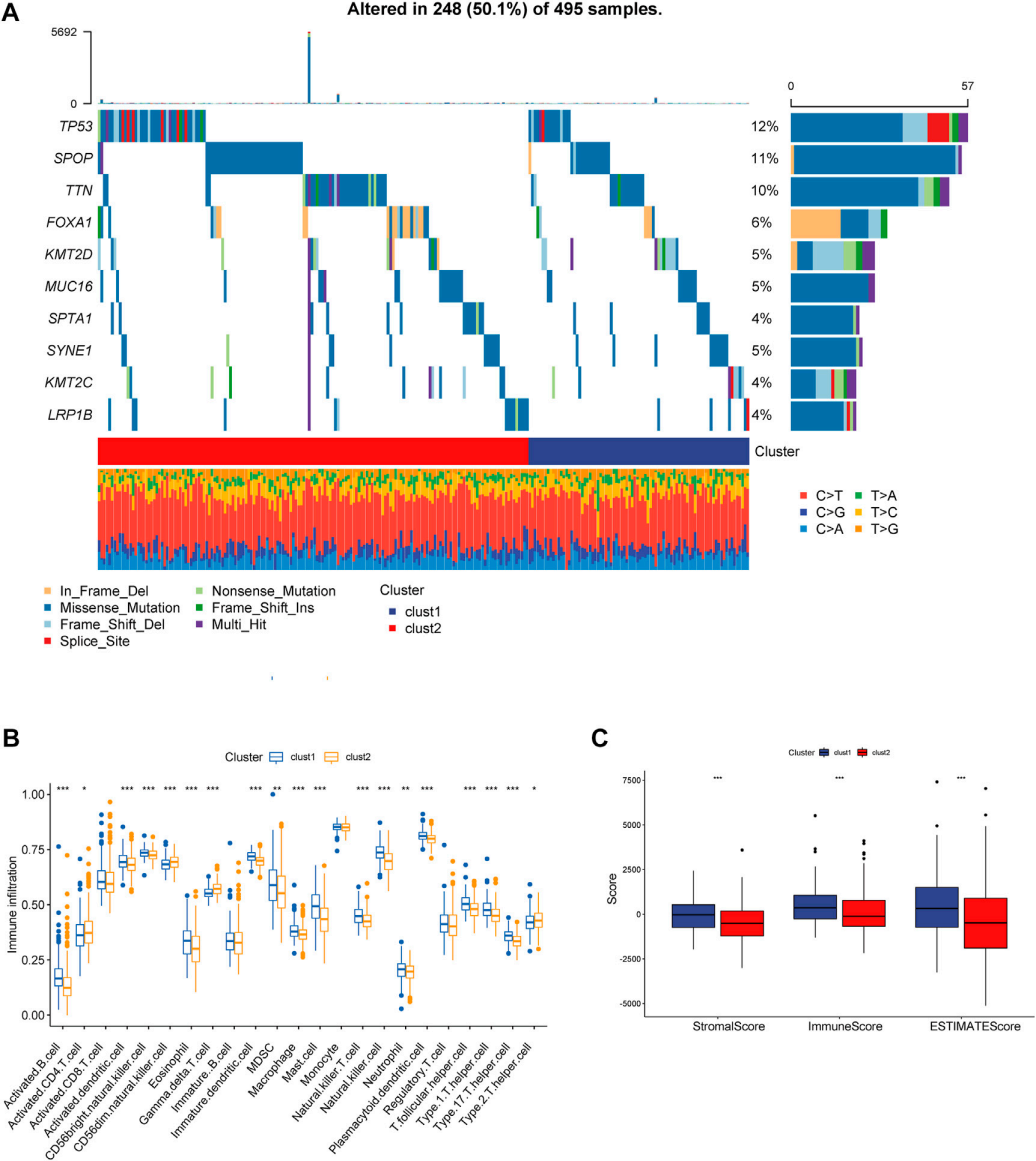
**(A)** Mutation information of the top 10 mutation genes is presented in the waterfall plot. **(B,C)** Box plot presenting the relative abundance of immune cells in the CRGs-related subtype.

We obtained simple nucleotide variation data from TCGA to explore differences in genomic mutations between CRs-related subtypes. The top 10 genes with the highest mutation frequencies are presented in Figure 4A; TP53, SPOP, and FOXA1 were more frequently mutated in cluster 2. Additionally, we evaluated the infiltration abundances of immune cells using the ssGSEA method to determine the association of each CR-related subtype with immune status. Patients in cluster 2 showed higher infiltration abundances of activated CD4 T-cell, CD56^dim^ natural killer cells, gamma-delta T-cell, and type 2 helper cells (Figure 4B). Furthermore, the ESTIMAT score further revealed that cluster2 had lower immune and stromal scores compared to the cluster1 (Figure 4C). Comprehensively, the results implied that CRs-related subgroups of prostate cancer can accurately indicate immunity status.

### Risk model construction

**FIGURE 5:**
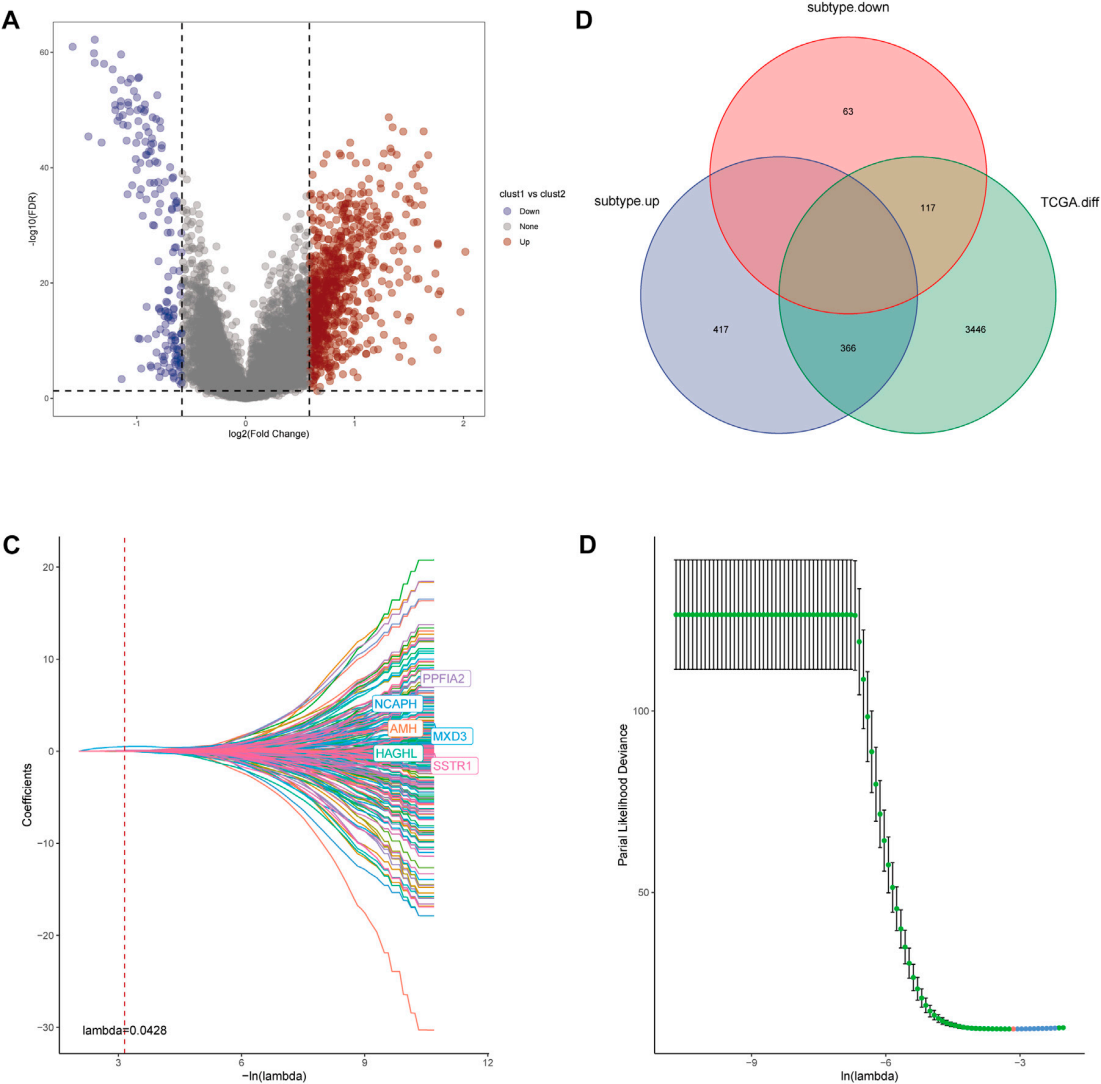
**(A)** Volcano diagram of DEGs between the two CRGs-related subtypes. **(B)** The Venn diagram demonstrated 483 genes obtained after the intersection of DEGs between the two subtype and DEGs between Prostate tumor and normal part in TCGA database. **(C,D)** LASSO Cox regression was utilized to construct the signature and the best log(λ) value was 0.0428.

For further investigation of mechanisms that contribute to the heterogeneity of CRs-related subtypes and application of these subtypes to clinical prediction and treatment, we firstly performed differential analysis of the two subtypes. We recognized 963 DEGs that were correlated with the two CRs-related subtypes (fold-change >1.5 and false discovery rate <0.05) (Figure 5A). The intersection of these DEGs with DEGs identified through comparisons of normal prostate and tumor tissue in TCGA yielded 483 DEGs (Figure 5B). Subsequently, univariate Cox analysis identified 261 genes that were correlated with RFS (supplementary Table S2). Next, we used lasso regression analysis to optimize the number of genes; we selected the six genes listed in Figure 5C. Finally, 4 genes including MXD3, SSTR1, AMH, and PPFIA2 were obtained based on the stepwise cox multivariate regression analysis. Then we constructed a 4 genes prognostic signature to predicate the RFS of PCa patients. Then, patients in the TCGA and Gene Expression Omnibus (GEO) cohorts were clustered into a high-risk group (HRG) and low-risk group (LRG) based on the following risk score (RS) formula:

Risk score = 0.796*MXD3+0.138*AMH+0.137*SSTR1+0.15*PPFIA2.

**FIGURE 6:**
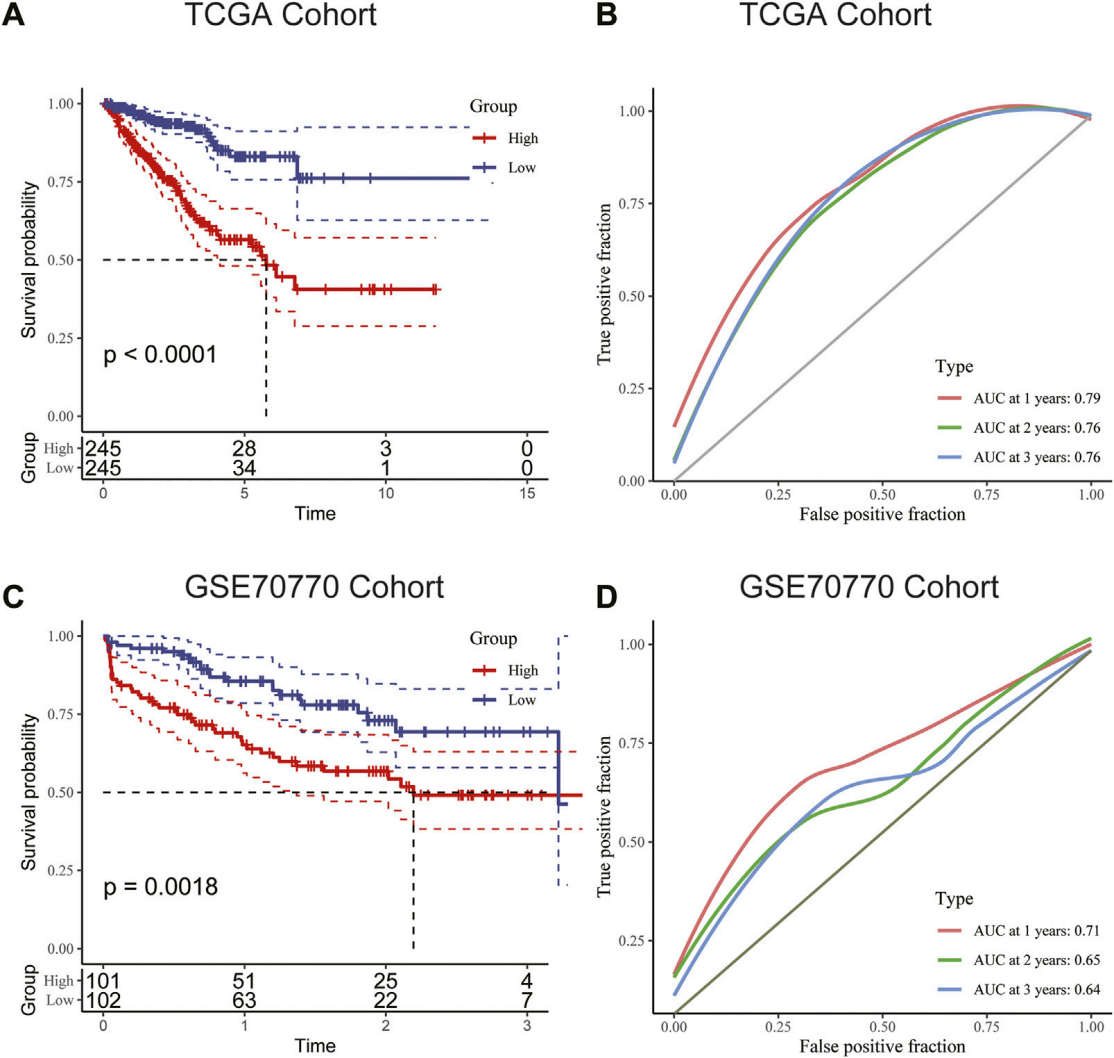
**(A)** Kaplan–Meier survival analysis suggested that patients in the high-risk group were more prone to recurrence in the training cohort. **(B)** The AUC curve plotted by ROC analysis of the signature revealed that the predictive performance of the signature was good. **(C)** Kaplan–Meier survival analysis suggested that patients in the high-risk group were also more prone to recurrence in the validation cohort. **(D)** The AUC curve plotted by ROC analysis of the signature was also presented in the validation cohort.

We used the median RS value as the cut-off for classifying patients into the HRG and LRG. K-M survival analysis indicated that the HRG exhibited a worse prognosis than the LRG in both the training and validation cohorts (Figures 6A, C). For additional assessment of risk model accuracy, we conducted receiver operating characteristic (ROC) analysis in both the training and validation cohorts. In the training cohort, the areas under the ROC curve of 1-year, 2-year, and 3-year RFS were 0.79, 0.76, and 0.76, respectively. In the validation cohort, the areas under the ROC curve of 1-year, 2-year, and 3-year RFS were 0.71, 0.65, and 0.64, respectively (Figures 6B, D).

### Construction of a nomogram containing RS and clinical characteristics

**FIGURE 7:**
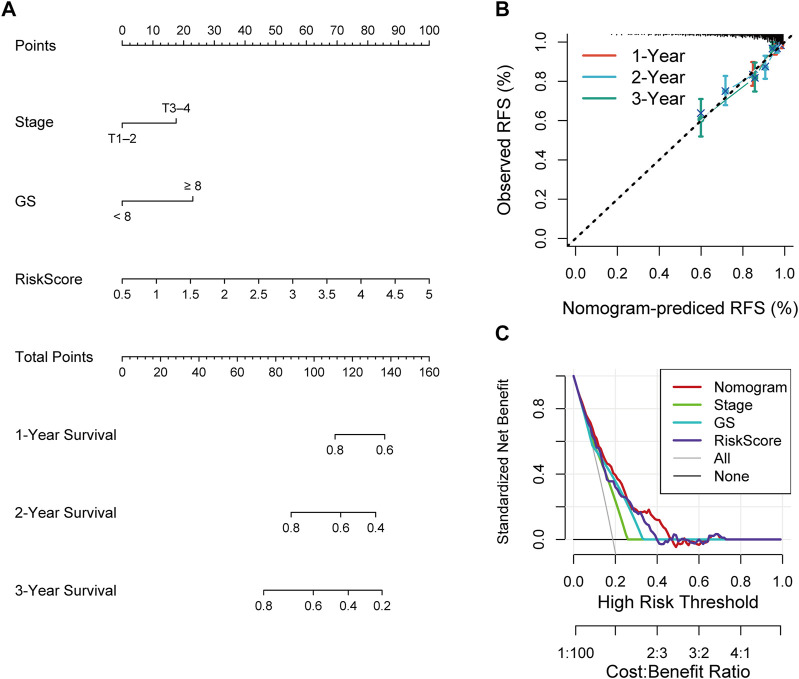
**(A)** Nomogram based on signature score, T-stage and GS predicting RFS of PCa patients from TCGA cohort. **(B)** The calibration curve of the nomogram. The y-axis is actual RFS and the x-axis is the nomogram-predicted RFS. **(C)** The Decision curve analysis (DCA) evaluating the clinical practicality of the nomogram.

Univariate and multivariate Cox analyses were conducted to assess the relationships of RFS with potential variables. Pathological T stage, RS and GS were identified as independent risk factors (Supplementary Figure S3A, B). Consequently, a nomogram with an integrated prognostic risk score model, pathological T stage and GS was constructed for RFS prediction in PRAD samples from prostate adenocarcinoma patients (Figure 7A). The calibration curves at 1, 2, and 3 years showed good linearity and suggested that the nomogram could accurately predict the RFS of PRAD patients (Figure 7B). Furthermore, the decision curve analysis (DCA) suggested that, compared with clinical parameters such as GS or pathological T stage, the nomogram showed superior net clinical benefit (Figure 7C).

### Distinct clinical characteristics and immune cell infiltration between the HR group and LR group

**FIGURE 8:**
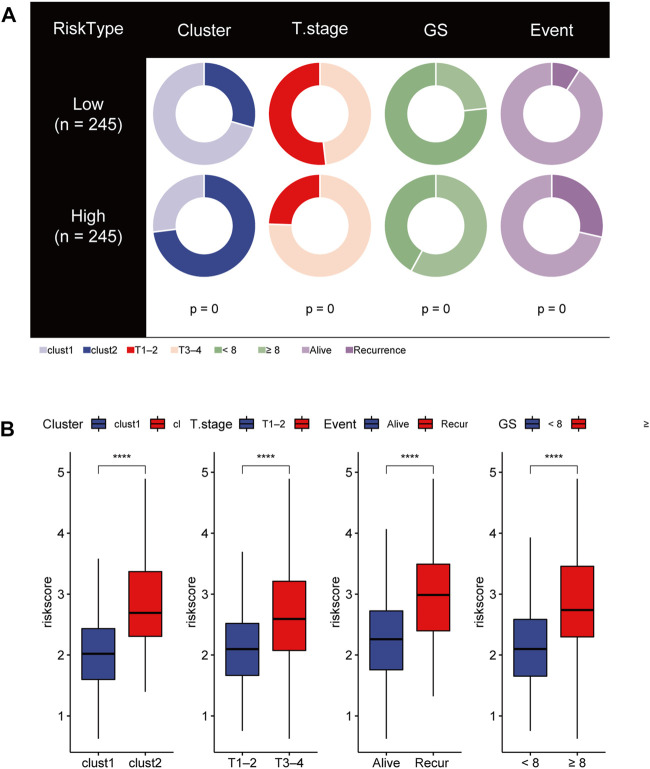
**(A)** Stratified proportion of clinical features of PCa patients in the HR group and LR group. **(B)** Association of the risk score with CRGs-related subtype and clinical characteristics including T stage, recurrence status and GS.

**FIGURE 9:**
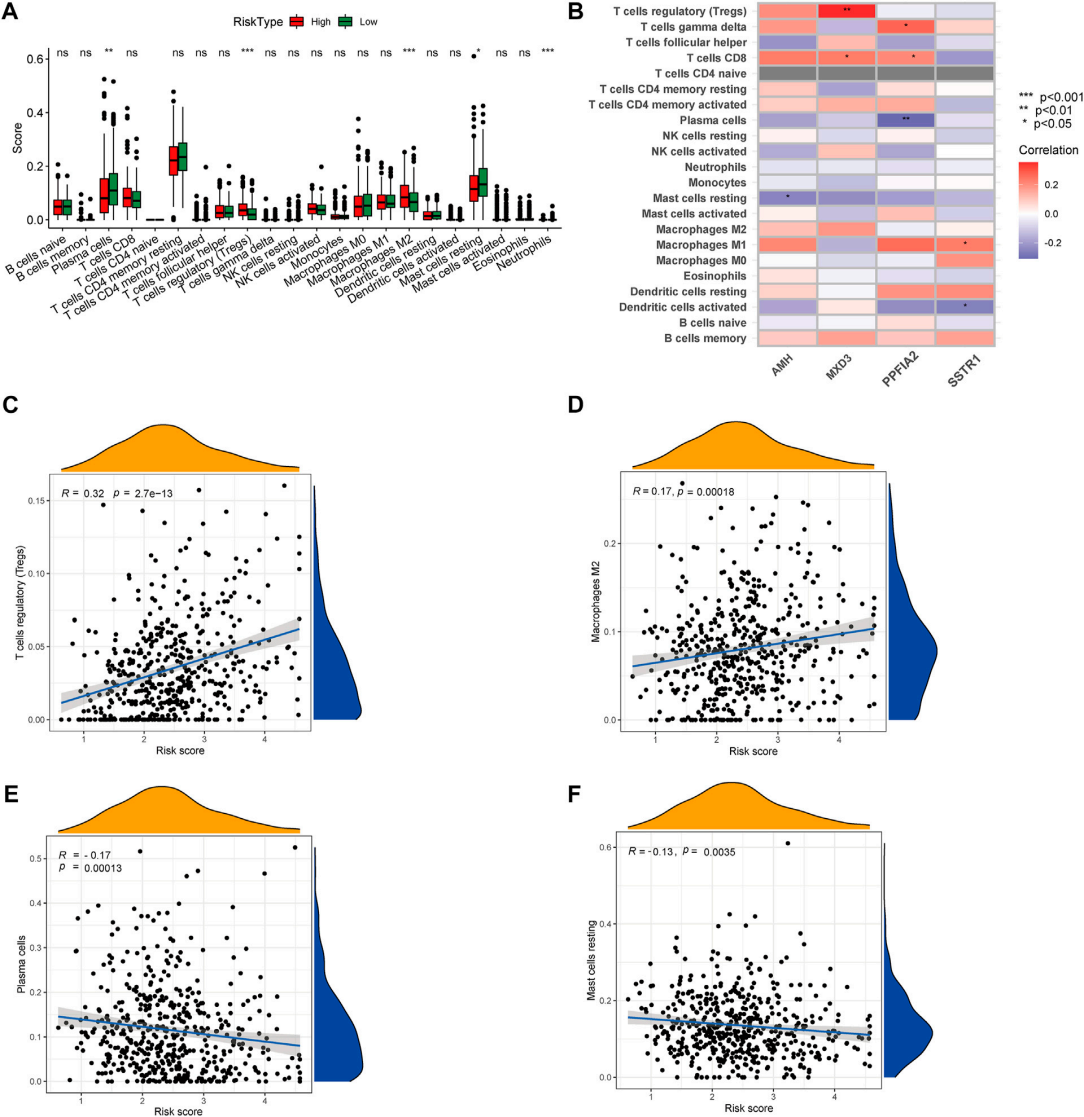
**(A)** CIBERSORT algorithm was used to estimate the relative abundance of tumor infiltrating immune cells between the different risk group. **(B)** Correlation analysis was performed to explore the relationship between core genes and infiltrating immune cells **(C,D)** The risk score was positively correlated with regulatory T-cell and M2 Macrophages **(E,F)** The risk score was negatively correlated with Plasma cells and resting mast cells.

To gain insights into the correlation between clinical features and risk model, we performed Chi-square test to study the distribution of patients in the HR group and LR group based on clinical characteristic including GS, T stage and clinical outcome. Results from the analysis provided the evidence that in the TCGA cohort HR group was significantly associated with higher GS, aggressive T stages and poor prognosis. Furthermore, patients in cluster 2 occupied a greater proportion of the HR group (Figures 8A, B). Additionally, to determine whether the signature score was associated with tumor immunity, we compared the numbers of distinct tumor-infiltrating immune cells between the two subtypes using the CIBERSORT algorithm. The results suggested that, compared with the LRG, the HRG contained more regulatory T-cell (*p* < 0.01) and more M2 macrophages (*p* < 0.01). In contrast, low-risk group had more plasma cells (*p < 0.01*) and more resting mast cells (*p < 0.05*) (Figure 9A). Notably, the signature score was positively associated with the enrichment scores of regulatory T-cell and M2 macrophages (Figures 9C, D). Conversely, the RS was negatively correlated with plasma cells and mast cells (Figures 9E, F). Previous studies demonstrated that high levels of infiltrating M2 macrophages and regulatory T-cell were correlated with biochemical recurrence (Andersen et al., 2021). This partly explain the poor prognosis of PCa patients in the high-risk group. Importantly, correlation analysis between risk-related genes and tumor-infiltrating immune cells revealed that MXD3 had the strongest positive correlation with the number of regulatory T-cell (Figure 9B).

### Identification of HRG-specific and LRG-specific molecular pathways and screening of small molecule drugs

**FIGURE 10:**
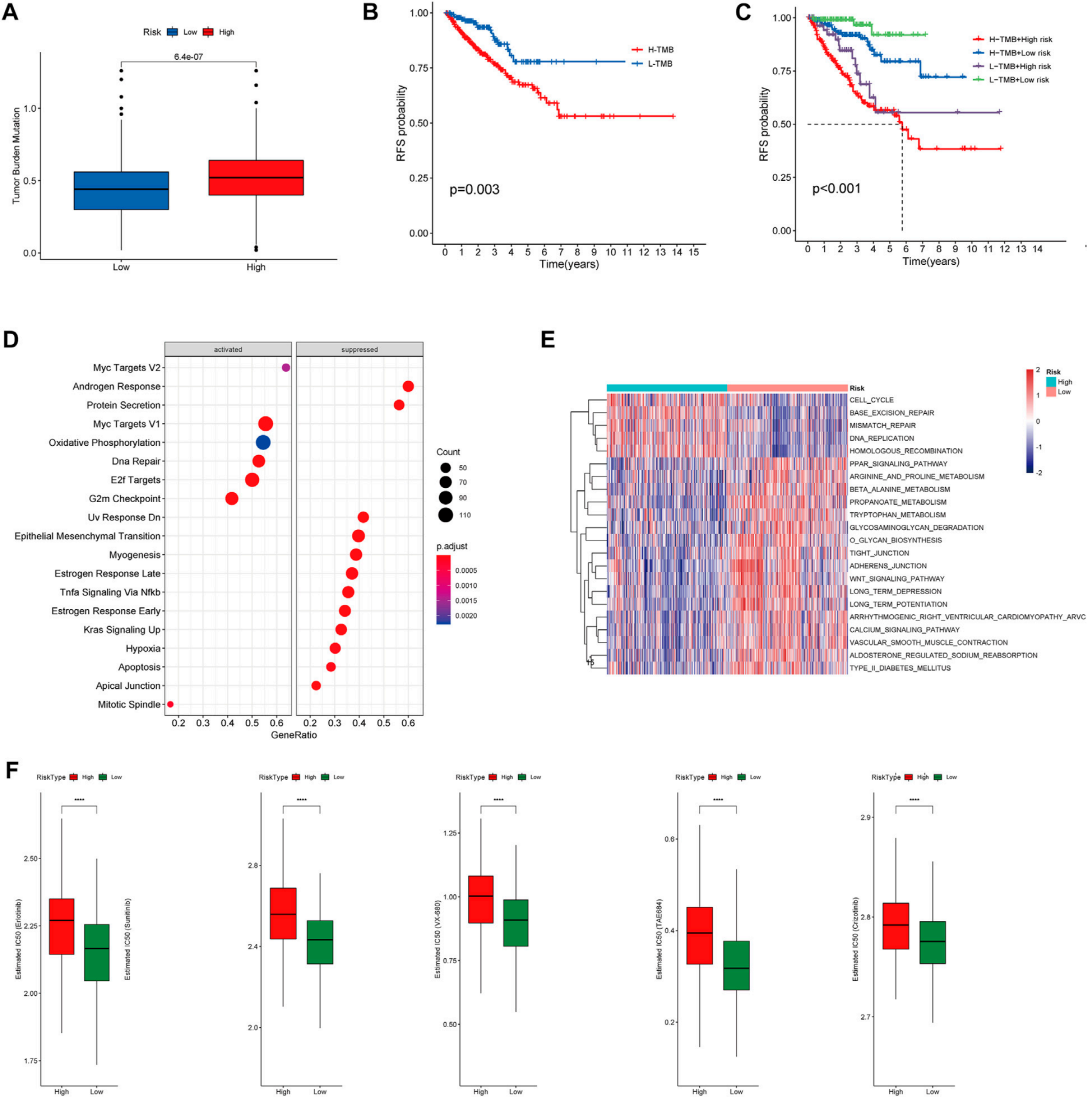
**(A)** Boxplot showing HR group possess higher TMB than LR group. **(B)** Kaplan–Meier survival analysis demonstrated that patients with high TMB had worse prognosis than patients with low TMB. **(C)** K-M plot also revealed that patients in the high-risk subgroup with high TMB had the Worst prognosis. **(D)** The heatmap of GSVA enrichment analysis showed the different signaling pathway between the HR group and LR group. **(E)** GSEA analysis demonstrated that the potential molecular mechanism activated in the HR group. **(F)** Box plots showing drugs including Erlotinib, Sunitinib, VX-680, TAE684 and Crizotinib are more sensitive in the high-risk group of patients.

To investigate correlation between the CRs-related signature and the mutational landscape, we calculated the tumor mutational burden (TMB). Compared with patients in the LRG, patients in the HRG exhibited higher TMBs (Figure 10A). Strikingly, K-M plots suggested that patients with higher TMBs were more likely to exhibit progression, compared with patients who had lower TMBs (Figure 10B). Furthermore, use of combined risk models showed that patients in the high-risk + high-TMB group exhibited the worst prognosis, according to K-M survival analysis (*p < 0.01*) (Figure 10C). These findings indicated that both the risk score and TMB can predict poor prognosis in Pca patients.

Gene set enrichment analysis (GSEA) suggested that patients in the HR and LR groups have different transcriptomic alterations. Gene Ontology terms enriched in the HRG were myc targets V2, myc targets V1, oxidative phosphorylation, DNA repair, E2F targets, and G2-M checkpoint (Figure 10D). Similarly, gene set variation analysis (GSVA) revealed that Gene Ontology terms enriched in the HRG were cell cycle, base excision repair, mismatch repair, DNA replication, and homologous recombination (Figure 10E). These findings indicated that the risk model was closely associated with cell cycle-related pathways or DNA repair-related pathways, which require the participation of multiple CRs. Because a high RS is associated with poor prognosis and multiple oncogenic signaling pathways contribute to progression in PCa patients, we used the pRRophetic package in R to explore the relationship between RS and potential targeted inhibitors. As shown in Figure 9F, high-risk score samples were more sensitive to Erlotinib, Sunitinib, VX-680, TAE684 and Crizotinib. These drugs may be used as alternative treatment for PCa progression in high-risk patients.

### MXD3 was essential for growth of PCa cells

**FIGURE 11:**
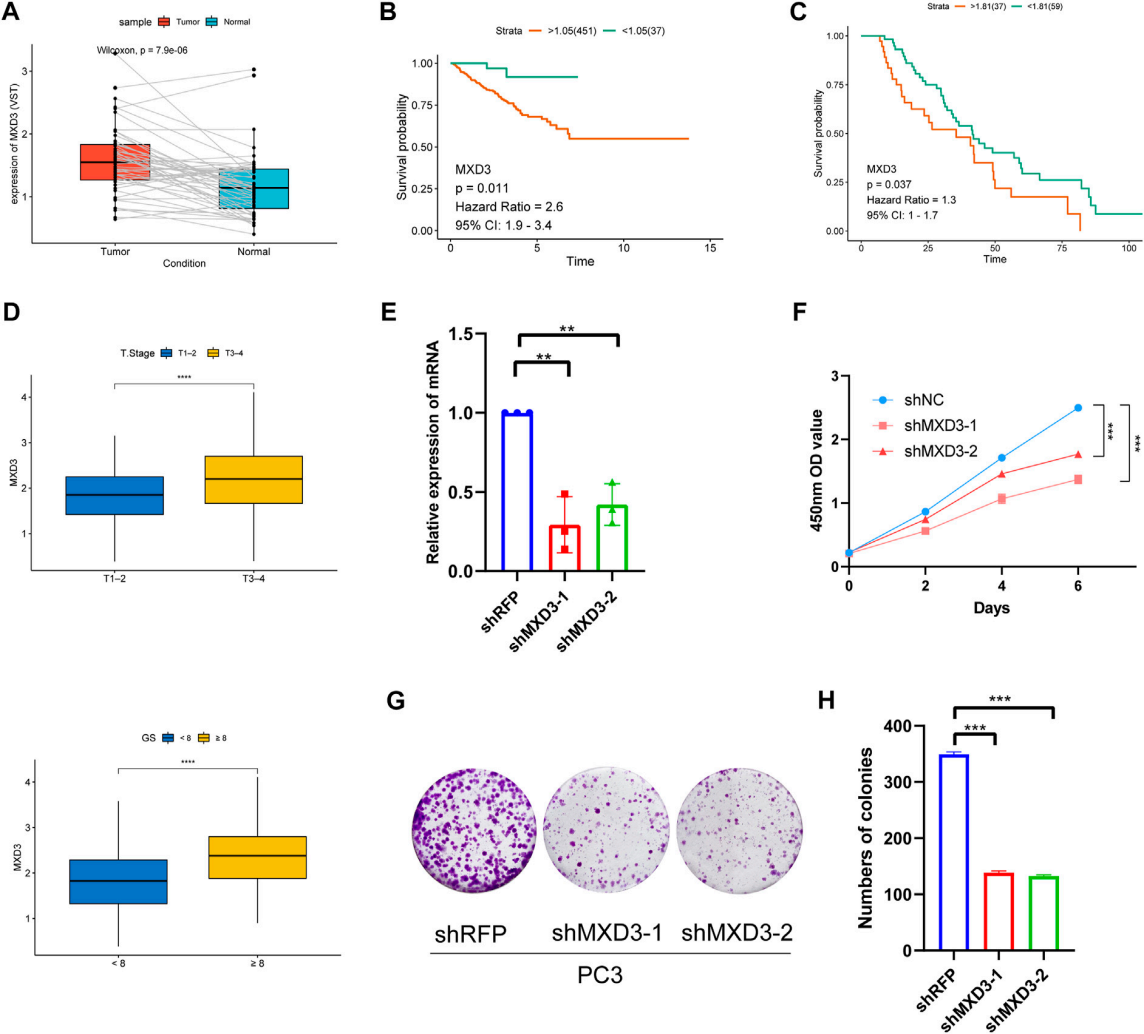
Clinical correlation analysis and *in vitro* experiment analysis of MXD3. **(A)** MXD3 expression in PCa paired tissues from the TCGA prostate adenocarcinoma (PRAD) dataset. **(B)** Kaplan-Meier curve of RFS (recurrence-free survival) in high and low MXD3 group in TCGA database. **(C)** Kaplan-Meier curve of OS (overall survival) for patients with high and low MXD3 expression in WCDT cohort. **(D)** Clinical correlation of MXD3 in TCGA prostate adenocarcinoma (PRAD) dataset. **(E)** PC3 cells were transfected with short hairpin RNAs targeting. The efficiencies of the shRNAs were verified by real-time PCR. **(F)** CCK8 assays revealed that knockdown of MXD3 remarkably reduce the cell viability. **(G)** Inhibition of MXD3 reduced the colony numbers in the colony formation assay. **(H)** The numbers of colonies in each group were counted. Each value means the mean ± standard deviation of three independent experiments. ***p < 0.001.

Because MXD3 is a hub gene with a central role in the PCa signature, we focused on MXD3 during *in silico* and *in vitro* analyses. Analysis of TCGA PCa data revealed that MXD3 was strongly upregulated in PCa tissue (Figure 11A). Kaplan-Meier analysis of TCGA data indicated that the level of MXD3 expression was significantly associated with RFS of Pca patients (Figure 11B). Moreover, K-M analysis of mCRPC patient data from the West Coast Prostrate Cancer Dream Team (WCDT) cohort (Quigley et al., 2018) showed that high expression of MXD3 was strongly associated with overall survival (Figure 11C). Additionally, we analyzed the differential expression level of the MXD3 in various pathological stages and Gleason score of Pca patients using TCGA data; the findings indicated that MXD3 is significantly upregulated in higher T stage and higher Gleason score groups (Figure 11D). For additional exploration of the biological function of MXD3 in PCa, we performed *in vitro* experiments to validate the oncogenic role of MXD3 in the PC3 PCa cell line. We silenced MXD3 in PC3 cells and used reverse transcription polymerase chain reaction to confirm MXD3 knockdown (Figure 11E). Cell proliferation was evaluated using the CCK-8 method, and the results suggested that MXD3 knockdown significantly reduced the growth of PCa cell (Figure 11F). Colony formation assays indicated that MXD3 inhibition considerably reduced the numbers of PC3 cell colonies (Figures 11G, H). Considering the role of AR in the progression of prostate cancer, we selected the C4-2 PCa cell for further validation. Similarly, MXD3 knockdown significantly reduced the growth of C4-2 (Supplementary Figure S4). Collectively, these results demonstrated that MXD3 is essential for growth of PCa cell.

## Discussion

The identification of distinct tumor molecular subtypes facilitates the rational use of new drugs and subsequent exploration of potential therapeutic targets. Heterogeneity has been studied in many cancers (e.g., breast cancer (Zardavas et al., 2015), lung cancer (Errico, 2014), gastric cancer (Shah and Ajani, 2010) and prostate cancer (Boutros et al., 2015). Heterogeneity in PCa prognosis is currently observed *via* risk stratification, which involves the prostate-specific antigen (PSA) level, T stage, Gleason score, the percentage of positive biopsy sores and age (Chang et al., 2014). Advances in molecular technology have revealed increasing evidence that CRs play significant roles in tumorigenesis and cancer progression. However, few studies have thoroughly analyzed CRs function in an effort to generate a molecularly heterogeneous model for PCa.

This study was conducted to identify a molecular subtype and constructed a new prognostic model based on CRs. Firstly, we screened 36 CRs-related genes in TCGA that were differentially expressed between prostate cancer tissues and normal tissues. Then, we performed univariate Cox regression analyses, which identified 18 CR-related genes that were associated with PCa RFS. Consequently, CRs-related subtypes were established based on these genes. Patients in cluster 2 experienced inferior clinical outcomes. This finding was confirmed by analysis of data in the GEO (GSE70770) cohort. Genetic alteration is one of the main mechanisms involved in the onset of PCa (Abida et al., 2019). Comparison of genomic mutations between the two subtypes revealed that mutations in genes such as TP53, SPOP, and FOXA1 were more common in cluster 2. Many studies have showed that mutations in TP53 contribute to the onset of metastatic PCa (Levine, 2020). TP53 and RB1 knockout models exhibit enzalutamide resistance and the upregulation of basal markers, neuroendocrine markers, and lineage-defining and stemness-related transcription factors, as well as the downregulation of luminal cell markers; these changes often suggest a more aggressive tumor and worse prognostic outcome (Levine, 2020). PCa-associated SPOP mutations reportedly confer resistance to BET inhibitors (Dai et al., 2017), which constitutes a new challenge in the treatment of PCa. FOXA1 mutations alter pioneering activity, differentiation and prostate cancer phenotypes (Adams et al., 2019). Alterations of biological behavior may lead to greater PCa malignancy. Additionally, analysis of 10 tumor abnormality-related pathways revealed that pathways associated with malignant phenotypes (e.g., cell cycle, MYC signaling, PI3K signaling, and TP53 signaling) exhibit greater activation in cluster 2 than in cluster 1. These alterations in key genes and signaling pathways imply the prognosis difference of the subtypes. Furthermore, analyses of immune cell relative abundances indicated that the numbers of activated B-cell, dendritic cells, CD56^bright^ natural killer cells, macrophages, and mast cells were greater in cluster 1 than in cluster 2. These changes in immune system and immune microenvironment may contribute to the observed prognostic differences.

To investigate the mechanisms contributing to heterogeneity in CRs-related subtypes and construct a signature for prediction of RFS in individual patients, we first analyzed the differences between cluster 1 and cluster 2. We identified four core genes (MXD3, SSTR1, AMH, PPFIA2) as independent risk factors based on the results of univariate Cox analysis, lasso regression, and multivariable Cox analysis. We finally established and validated a model, in which low and high risk were classified according to RS, for prediction of individualized clinical prognostic outcome. Survival analyses revealed that the model demonstrated good predictive ability. We then explored the relationships of RS with clinical characteristics of PCa; we observed significant differences between the HRG and LRG in terms of T stage, GS, and recurrence status. Furthermore, we found that the signature was positively associated with the numbers of infiltrating M2 macrophages and regulatory T-cell, which contribute to the biochemical recurrence of PCa. TMB, defined as the number of somatic mutations per megabase of interrogated genomic sequence (Sha et al., 2020), is emerging as a predictive biomarker in solid tumors; it can be used to predict clinical responses of many cancers to immune checkpoint inhibitor treatment (Chan et al., 2019). Our study showed that the risk score has good prognostic value, regardless of whether it is used in combination with the TMB. Compared with other risk model with potential to predicate prognosis of PCa patients (Liu et al., 2018; Martini et al., 2019), CRs signature comprehensively evaluated the differences of gene mutation and immune profile between high and low risk groups in risk model; and predicated potential drug targets in high-risk PCa patients. Importantly, we selected the hub gene in CRs signature for further biological verification.

The risk score is computed with four genes including MXD3, SSTR1, AMH and PPFIA2. Their function contributing to the development of PCa remains to be explored. We confirmed that the downregulation of MXD3 significantly suppressed the proliferation of PCa cells *in vitro*. PPFIA2 (liprin-α2) is an important component of R2TP, an HSP90 co-chaperone (Maurizy et al., 2018). In cancer cells, HSP90 facilitates the function of numerous oncoproteins (Trepel et al., 2010). Analysis of the relationship between PPFIA2 and HSP90 indicated that PPFIA2 may affect the biological behavior of PCa by stabilizing HSP90, although the mechanism has not yet been clarified. PPFIA2 is used as a prognostic factor in the early diagnosis of PCa (Leyten et al., 2015), consistent with our findings. MXD3, a member of the MXD family, plays pivotal roles in cell cycle progression and cell proliferation; it is regarded as an onco-immunological biomarker (Wu et al., 2021). Moreover, pan-cancer analysis discovered that MXD3 interacted with gene ancestry (GA) and exacerbated observed survival disparities (Lee et al., 2022). Besides, MXD3 was reported to be a potential therapeutic target in pre-B cell acute lymphoblastic leukemia (Satake et al., 2014). However, the function of MXD3 in prostate cancer requires further investigation. Somatostatin receptor 1 (SSTR1) belongs to the G protein coupled receptor family and have a wide expression pattern in solid tumors (Theodoropoulou and Stalla, 2013). SSTR1 have been reported to be the most prominent candidates of biomarkers associated with aggressive prostate cancer phenotype (Kosari et al., 2008). In addition to that, SSTR1 plays a significant role in the onset and progression of prostate cancer. Depending on the cell system and extracellular environment, activation of the mitogen-activated protein kinase (MAPK) pathway can also halt cell growth, thereby promoting cell differentiation. PCa may transform into neuroendocrine prostate cancer (NEPC), which exhibits more aggressive clinical behavior and a poor prognosis. Although neuroendocrine PCa can arise *de novo*, most PCa patients are diagnosed with standard prostatic adenocarcinoma and receive hormone therapy before the onset of neuroendocrine PCa, leading to the term “treatment-related neuroendocrine PCa” (Beltran et al., 2012; Tagawa, 2014). Anti-Mullerian hormone (AMH) is also reportedly associated with PCa prognosis.

GSEA and GSVA analysis revealed enhanced activation of the cell cycle pathway and DNA repair pathway in the HR group. Disruption of cell cycle regulatory mechanisms can lead to uncontrolled growth of normal cells. Many factors can regulate cell proliferation *in vivo*, including CRs. DNA damage repair genes may confer an increased risk of early-onset PCa (Attard et al., 2016). However, DNA repair pathway dysfunction may contribute to resistance to DNA-damaging chemotherapy and radiotherapy (Curtin, 2012). Additionally, we also applied “pRRophetic” R package to identify the sensitive drugs in the HR group. The results suggested that drugs including Erlotinib, Sunitinib, VX-680, TAE684 and Crizotinib possess higher IC50 in HR group. However, these results warrant further research, both *in vivo* and *in vitro*.

Although we identified stable molecular subtypes and successfully developed a powerful prognostic signature, this study had limitations that should be addressed in future research. Firstly, we constructed and validated the signature utilizing retrospective data from the TCGA and GEO database. Prospective real-world data are needed to assess clinical utility of the molecular signature. Second, there is a need for further *in vitro* and *in vivo* experiments to explore underlying mechanisms correlated with the CR-related subtypes and risk model.

## Conclusion

Overall, this study identified CRs-related subtypes in prostate cancer and constructed a prognostic signature based on CRs-related subtypes. The clinical characteristics, gene mutation status, immune profile and drug sensitivity between the two subtypes and different risk groups were also investigated. The molecular signature may provide evidence for clinical judgement of individual patient prognosis and personalized treatment.

## Data Availability

The original contributions presented in the study are included in the article/Supplementary Material, further inquiries can be directed to the corresponding authors.
