# Supplementary material for: Identification of molecular subtypes and a prognostic signature based on chromatin regulators related genes in prostate cancer
Source: Front Genet. 2023 Jan 10;13:1110723. doi: 10.3389/fgene.2022.1110723 (PMC9871366; doi:10.3389/fgene.2022.1110723)
Supplement: Supplementary file 1 [file DataSheet2.PDF]

	p.value	HR	Low 95%CI	High 95%CI
RBFOX3	0.0001332	0.6655799	0.5401287	0.8201685
FAM107A	0.0001298	0.6971442	0.5795337	0.8386225
SRD5A2	6.88E-06	0.7104119	0.612068	0.8245573
JPH4	0.0004388	0.7258063	0.6070543	0.8677885
C2orf88	0.0003575	0.5828199	0.433307	0.7839224
HIF3A	0.0171077	0.6885707	0.50669	0.9357391
COL4A6	0.0038953	0.7441688	0.6088855	0.9095097
NECAB1	0.0176144	0.6605881	0.4690757	0.9302906
PYGM	0.0004348	0.7164418	0.594975	0.8627067
PGM5	0.0003175	0.7770111	0.6773008	0.8914005
MPP2	0.0133399	0.7252969	0.5623909	0.9353915
RND2	0.0103766	0.6847266	0.5125493	0.9147422
CSRP1	0.0034458	0.727929	0.5884032	0.90054
NRG2	0.0085569	0.6039353	0.4146978	0.8795269
HSPB8	0.0006416	0.7817573	0.6786938	0.9004715
MYOCD	0.0002787	0.7010589	0.5788576	0.8490578
TENT5B	0.0006289	0.7269826	0.6055325	0.8727916
SYNM	0.001364	0.7900714	0.68396	0.9126454
TMEM35A	9.28E-05	0.7444033	0.6419878	0.863157
CCDC178	0.0018663	0.4983806	0.3213693	0.7728903
IP6K3	0.0001895	0.6386093	0.5046214	0.8081739
JPH2	0.0088164	0.7813354	0.6495968	0.9397907
ACOX2	0.0038543	0.724779	0.5826311	0.9016075
MYL9	0.0145432	0.8225258	0.7032205	0.962072
SMOC1	0.0036398	0.8124057	0.7062471	0.9345215
KCNMB1	0.0056678	0.7758578	0.6481778	0.9286887
MYH11	0.0007152	0.8062582	0.7117055	0.9133725
ASB2	0.0180421	0.7856884	0.6433264	0.9595538
GNAL	0.0096814	0.6968595	0.5300405	0.9161812
GSTM5	0.0134927	0.7461036	0.5914041	0.9412693
PAK3	0.0053054	0.5635511	0.3765581	0.8434019
PRIMA1	0.0286409	0.7864476	0.6342052	0.9752361
LDB3	0.0004185	0.7239348	0.6050047	0.8662439
PLA2G2C	0.0004119	0.4606804	0.2996629	0.7082172
UBXN10	4.09E-05	0.5532121	0.4169163	0.7340649
PPARGC1A	0.0006866	0.5352326	0.373097	0.7678271
COL23A1	0.0111533	0.713555	0.5498332	0.9260277
ATP1A2	0.0001078	0.7436553	0.640127	0.8639272
CNN1	0.0040606	0.8187996	0.7144238	0.9384246
SCN2B	0.0072355	0.5772511	0.3865637	0.8620025
PRICKLE2	0.0203007	0.6735942	0.4824735	0.9404227
CAV1	0.0320475	0.8090889	0.6666267	0.9819961
MEIS2	0.0236308	0.7897446	0.6437302	0.9688788
ACTG2	0.0013602	0.8156059	0.7199679	0.923948
CA14	0.000459	0.5677027	0.4135922	0.7792371
FLNC	0.0041107	0.8219711	0.7189548	0.9397482
EDN3	0.0010741	0.6448763	0.4957857	0.8388008
ASPA	0.0040954	0.5914802	0.4132701	0.8465379
RASL12	0.0082154	0.7832932	0.6535287	0.9388238
ZNF185	0.0022126	0.7339719	0.602072	0.894768

GSTM4	0.0048125	0.6715555	0.5091797	0.8857123
C8orf88	0.0096138	0.7229883	0.5655955	0.92418
CPNE6	0.0017321	0.6175023	0.4567221	0.834882
CLIC6	0.0097465	0.7686728	0.6296508	0.9383899
ALDH1A2	0.0006278	0.7039832	0.575684	0.8608758
B3GALT2	0.0001184	0.5824948	0.4423696	0.7670062
OGN	0.0030504	0.7917093	0.67836	0.9239984
ROR2	0.026056	0.8158905	0.6820293	0.9760243
ZNF516	0.0316944	0.782938	0.6262804	0.9787819
EYA4	0.0014462	0.5917812	0.4285157	0.8172513
FAXDC2	0.0311591	0.7873668	0.6335081	0.978593
PCDH7	0.0144872	0.7716002	0.6267897	0.9498671
LMOD1	0.0135836	0.8204105	0.7010703	0.9600655
RAB9B	0.0046264	0.6893228	0.5328403	0.8917606
PDZRN4	0.0035818	0.7442735	0.6101331	0.9079051
LGI3	0.0095982	0.5940489	0.4005551	0.8810127
CCBE1	0.0001118	0.4602181	0.3104399	0.68226
ITIH5	0.0346168	0.7777901	0.6160561	0.9819844
ANGPT1	0.0011204	0.7208548	0.5920276	0.8777151
AOX1	0.0022101	0.7685844	0.649361	0.9096973
DES	0.0111931	0.8532	0.7547027	0.9645523
P2RX1	0.0179282	0.7878227	0.6466526	0.9598116
RPE65	0.0002469	0.5122369	0.358196	0.7325225
FLNA	0.0142263	0.8235751	0.7051934	0.9618296
STAC	0.0015636	0.7222771	0.5903893	0.8836275
ACSS3	0.0123432	0.6395064	0.4505704	0.9076684
IGSF1	0.0007646	0.4792654	0.3122786	0.7355461
SHISAL1	0.0045853	0.720001	0.5737125	0.9035909
GLIS1	0.0434064	0.7909059	0.6298928	0.9930772
TMEM252	0.0004917	0.7186304	0.5967734	0.8653697
SYNPO2	0.0024866	0.8045123	0.6987544	0.9262769
SNAI2	0.0416675	0.8246077	0.6849443	0.9927491
CLVS2	0.0164434	0.6607977	0.4710437	0.9269917
ADRA1D	0.0004761	0.6377086	0.4954724	0.8207769
SLC2A5	0.0162039	0.8068159	0.6772944	0.9611062
C3orf70	0.0065599	0.7231266	0.5724108	0.9135257
CHRDL1	0.0032274	0.8205489	0.7193546	0.9359785
KIAA1210	0.0037624	0.7764399	0.6542914	0.9213921
ATP2B4	0.0063813	0.782103	0.6554877	0.9331756
RNF112	0.0497828	0.7654761	0.5861022	0.9997465
ATCAY	0.0156618	0.7419337	0.5824119	0.9451484
PLIN4	0.0279562	0.7982851	0.6529923	0.9759061
WFDC2	0.0063327	0.8637637	0.7775554	0.9595301
ACTC1	0.0018617	0.8255153	0.7315914	0.9314973
JAZF1	0.0458386	0.7793419	0.6101793	0.9954022
ACTA2	0.0306154	0.8348277	0.7087965	0.9832685
MAMLD1	0.0062555	0.6525155	0.4804825	0.8861436
FGF10	0.0075558	0.6875743	0.5223472	0.9050655
TNS1	0.0226949	0.8266277	0.7017478	0.9737307
NDP	0.0022186	0.7012698	0.5586632	0.8802787
MYLK	0.0044763	0.8249866	0.7224953	0.9420171

VIT	0.0015442	0.6117666	0.4513133	0.829265
PTGIS	0.0035231	0.7690055	0.6446292	0.9173794
CRYAB	0.0260853	0.805849	0.6663033	0.9746202
LGR6	0.0414935	0.7970107	0.6408074	0.9912901
IRAG1	0.0320386	0.8161657	0.6778393	0.9827203
ANO5	0.0013808	0.6590224	0.5104237	0.8508823
CES1	0.0332994	0.8418275	0.7183988	0.9864625
ANGPTL1	0.0003325	0.6618303	0.5282564	0.8291794
ADRA1A	0.0020072	0.6535777	0.4990108	0.856021
FRMD6	0.010353	0.770611	0.6314279	0.9404736
SORBS1	0.0112443	0.8193823	0.7024221	0.9558176
DPT	0.0018698	0.7654038	0.6467269	0.9058585
GPR161	0.0383511	0.7689877	0.5997352	0.9860055
MSRB3	0.0373979	0.8233796	0.6856843	0.9887262
FHL1	0.0311614	0.8416466	0.7195083	0.9845183
PPP1R1A	0.0285408	0.7532995	0.5845842	0.9707072
NEXN	0.0341481	0.823278	0.6877115	0.9855682
PDK4	0.0161034	0.839498	0.7280135	0.9680547
CAVIN2	0.0115889	0.7781641	0.6404699	0.9454611
PGAP4	0.0488792	0.8193763	0.6720351	0.9990214
LIX1	1.74E-05	0.4472417	0.3098205	0.6456164
SLC5A8	0.0005767	0.5950997	0.442842	0.7997066
CAPN6	0.0020137	0.7670964	0.648289	0.9076767
SGCG	0.0100736	0.6945098	0.5261283	0.9167798
TENM3	0.0363725	0.7316267	0.5459957	0.9803696
TGFBR3	0.0248302	0.7726582	0.6168144	0.9678775
GGT6	0.0289159	0.837262	0.7139301	0.9818995
ARHGAP20	0.0226185	0.7197783	0.5425406	0.9549162
SLC8A1	0.0263518	0.7594543	0.5957272	0.9681794
KCNAB1	0.0023738	0.7006945	0.5570626	0.8813603
CCK	0.0208168	0.8618054	0.7596901	0.9776466
MAMDC2	0.0403025	0.7812738	0.617087	0.9891454
NDRG2	0.0267378	0.7485989	0.5794202	0.9671741
NLGN3	0.0379307	0.786465	0.6268604	0.9867064
SPON1	0.0295256	0.8185504	0.6834993	0.9802859
NCAM1	0.0488406	0.7930548	0.6296779	0.9988218
RSPO3	0.0474073	0.7895493	0.6250753	0.9973008
SPOCK3	0.0010685	0.7758614	0.6664388	0.9032502
CFL2	0.0393231	0.7602274	0.5857495	0.9866772
PGR	0.0237969	0.7213716	0.5434632	0.9575202
SLC51B	0.0012979	0.6295911	0.4749121	0.8346491
HFE	0.0310875	0.7563902	0.5868329	0.9749388
P2RX2	0.00565	0.6971359	0.5399467	0.900086
TMEM132C	0.0158162	0.6857806	0.5048164	0.9316161
MAL	0.0373692	0.7963551	0.6426834	0.9867712
AHNAK2	0.0342932	0.7956577	0.6438719	0.9832253
RSPO2	0.0006544	0.6740685	0.5372678	0.8457018
MAP1A	0.0302368	0.7744016	0.614525	0.9758722
PDE5A	0.0070357	0.7964917	0.6750227	0.9398187
KCNMA1	0.0394269	0.7730263	0.6050659	0.9876109
DPYS	0.0176552	0.7988857	0.6636432	0.9616888

PLN	0.0471743	0.8371964	0.7024537	0.9977849
PLP1	0.0279233	0.7734481	0.6151221	0.9725256
BMPER	0.0010596	0.5841537	0.4234167	0.8059094
SERPINF2	0.0043535	0.7747038	0.6500242	0.923298
LRCH2	0.0108823	0.7607557	0.6163702	0.9389636
FOXI1	0.0159221	0.747706	0.5902969	0.9470899
PENK	0.0185507	0.7683165	0.6169607	0.9568036
JAM3	0.032447	0.8182253	0.6808213	0.9833602
TBX5	0.0022766	0.6602386	0.5057055	0.8619938
SERPINB5	0.0215169	0.8218433	0.6952445	0.9714948
PLCL1	0.0080003	0.7422127	0.5954485	0.9251508
CPED1	0.0264686	0.8232366	0.6932974	0.9775293
CD38	0.0002287	0.820303	0.7382773	0.9114421
HSD11B1	0.0164056	0.7832691	0.6416006	0.9562187
OLFM4	0.0195202	0.9147032	0.8487612	0.9857682
ZNF750	0.0357639	0.8472083	0.7257208	0.9890332
NDNF	0.0106987	0.8009347	0.6754138	0.9497827
SCGB1A1	0.0346338	0.8789371	0.7797673	0.9907192
CD177	0.0154436	0.9020623	0.829869	0.980536
CDO1	0.0457528	0.8771369	0.7712751	0.9975289
PLA2G4D	0.0476938	0.8123426	0.6612958	0.9978902
PTGS2	0.0362477	0.8680042	0.7602959	0.9909712
PGC	0.0159502	0.9024156	0.8301206	0.9810067
BIRC5	5.16E-07	1.6098332	1.3367522	1.9387011
UBE2C	1.16E-07	1.5644594	1.3258459	1.8460162
CDC20	3.60E-08	1.7055758	1.4105304	2.0623368
CENPA	5.69E-08	1.7517245	1.4307334	2.1447314
TPX2	6.15E-07	1.627353	1.343876	1.9706266
KIF4A	2.92E-07	1.7134663	1.3947522	2.1050095
SKA3	1.05E-05	1.6687458	1.3288751	2.0955412
PIMREG	8.64E-08	1.8975333	1.5008491	2.3990639
CDCA5	6.71E-09	1.9040299	1.5315525	2.3670947
KIFC1	7.66E-07	1.7208954	1.3876886	2.1341106
TROAP	4.89E-10	1.9791951	1.5962627	2.4539903
CDC25C	3.86E-08	2.181355	1.6517992	2.8806828
GTSE1	1.03E-07	1.8896162	1.4947525	2.3887895
HJURP	1.17E-06	1.6397604	1.3433186	2.0016207
AURKB	9.48E-08	1.7316045	1.4153732	2.1184903
NEK2	2.12E-06	1.5690302	1.3024511	1.8901712
SPC24	1.29E-08	1.9278902	1.5376136	2.4172267
KIF20A	5.02E-06	1.5997099	1.30742	1.9573449
DLGAP5	1.10E-05	1.615926	1.3046359	2.001491
PLK1	3.39E-09	1.9753532	1.5762351	2.4755318
MELK	1.06E-05	1.5945	1.2955773	1.9623917
RRM2	7.61E-06	1.571919	1.2894828	1.9162174
CDCA3	6.51E-07	1.8952833	1.473288	2.4381512
PBK	4.26E-05	1.4861308	1.2293144	1.7965988
IQGAP3	1.83E-07	1.8248535	1.4557006	2.2876203
EZH2	3.58E-06	2.0522804	1.5142154	2.7815427
HMMR	8.20E-05	1.5361896	1.2406806	1.9020839
SKA1	0.0002015	1.6832542	1.2791021	2.2151044

BUB1	1.63E-06	1.725941	1.3808144	2.1573298
TOP2A	6.38E-05	1.4026102	1.188256	1.6556324
CENPF	2.13E-05	1.5508558	1.2667894	1.8986217
SPC25	6.00E-06	1.7464941	1.3718432	2.2234623
MYBL2	1.46E-06	1.5293048	1.286531	1.8178909
CEP55	3.19E-05	1.5786192	1.2730558	1.9575251
KIF18B	4.56E-08	1.9108485	1.5150231	2.41009
PKMYT1	1.12E-07	1.9251669	1.5114381	2.4521465
TEDC2	1.09E-06	2.0142925	1.5200051	2.6693163
DEPDC1B	0.0011067	1.4828877	1.1702941	1.8789772
CDC45	1.41E-07	1.7829802	1.4375758	2.2113744
CCNA2	1.96E-05	1.5823063	1.281749	1.9533412
NCAPH	2.14E-07	1.7862939	1.4346615	2.2241106
FOXM1	1.89E-06	1.643151	1.339556	2.0155524
NCAPG	5.66E-05	1.6115348	1.277506	2.0329019
ESPL1	7.09E-08	2.1356981	1.6206359	2.8144547
EXO1	2.65E-07	1.89789	1.48697	2.4223666
BUB1B	7.28E-05	1.6428557	1.2855181	2.099523
MKI67	7.88E-05	1.5062186	1.2290756	1.8458543
FAM111B	0.0011251	1.328247	1.1196909	1.5756491
CENPM	0.0055686	1.3559033	1.0933036	1.6815766
SHCBP1	2.61E-06	1.922932	1.4639649	2.5257897
ASPM	2.54E-05	1.8152474	1.3753604	2.3958254
PPP1R14B	0.006273	1.5525327	1.1325158	2.1283217
GGCT	0.0053371	1.4672615	1.1203832	1.9215357
CBX2	6.44E-05	1.5322197	1.2428783	1.8889197
IQANK1	0.0236457	1.3337219	1.0392967	1.7115556
MXD3	8.29E-11	2.5545285	1.9248845	3.3901336
PRR7	1.54E-06	1.7162989	1.3769799	2.1392337
TMEM132A	0.0004907	1.5043954	1.1957373	1.8927281
ARHGDIG	0.0001388	1.3502581	1.1569994	1.5757976
CCDC78	5.63E-07	1.4723606	1.2653207	1.7132777
HES6	0.0075998	1.2678704	1.0651049	1.5092366
RHPN1	2.66E-05	1.6312438	1.298271	2.0496154
RGS11	1.04E-07	1.6484994	1.3711755	1.9819127
MMP11	4.64E-06	1.4103271	1.2173722	1.6338656
FGFRL1	0.0396911	1.2620568	1.0110343	1.5754038
HAGHL	1.13E-07	1.6334979	1.3626343	1.9582037
ZMYND10	0.0118009	1.2302869	1.047004	1.4456544
ISX	0.0374204	1.2096303	1.0111435	1.44708
FNDC10	0.0056063	1.3465615	1.0909089	1.6621259
AMH	8.79E-08	1.5193684	1.3035057	1.7709782
HOXC6	0.0287157	1.2352943	1.0222113	1.4927952
MAPK8IP2	0.0005774	1.4484956	1.1729699	1.7887411
AK5	0.0020276	1.2690906	1.0908537	1.4764501
MNX1	0.0342459	1.2131187	1.0144481	1.4506971
HOXC4	0.0001407	1.4657303	1.2038189	1.784625
SSTR1	3.96E-05	1.2721983	1.1342028	1.4269834
PDIA2	0.0008943	1.20137	1.0781161	1.3387146
COL10A1	0.0046253	1.1948653	1.0563525	1.3515404
GNG13	0.0116365	1.238089	1.048813	1.4615229

CPNE7	0.0334684	1.1683167	1.0122519	1.3484429
APLN	0.0141569	1.2454989	1.0451326	1.4842783
B4GALNT4	5.29E-06	1.5453969	1.2813292	1.863886
DNAH8	0.0132643	1.1502534	1.0296316	1.2850062
CTHRC1	0.0002012	1.3375726	1.1474112	1.5592496
COMP	0.002093	1.1882724	1.064614	1.3262941
PPFIA2	5.17E-06	1.2918136	1.1571211	1.4421847
