# Supplementary figures and images for: Identification of molecular subtypes and a prognostic signature based on chromatin regulators related genes in prostate cancer
Source: Front Genet. 2023 Jan 10;13:1110723. doi: 10.3389/fgene.2022.1110723 (PMC9871366; doi:10.3389/fgene.2022.1110723)

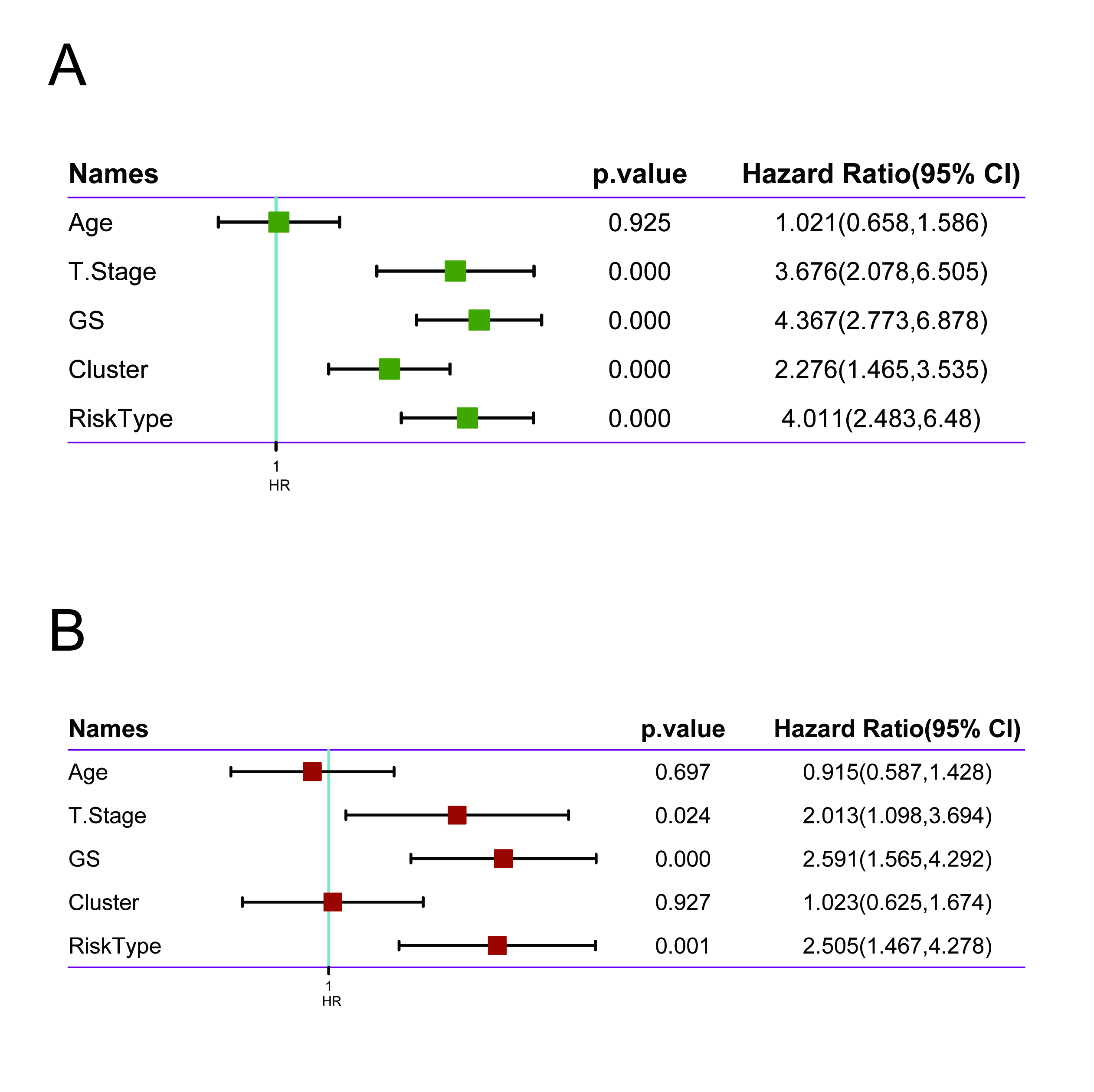

Supplement: Supplementary file 2 [file Image3.TIF]

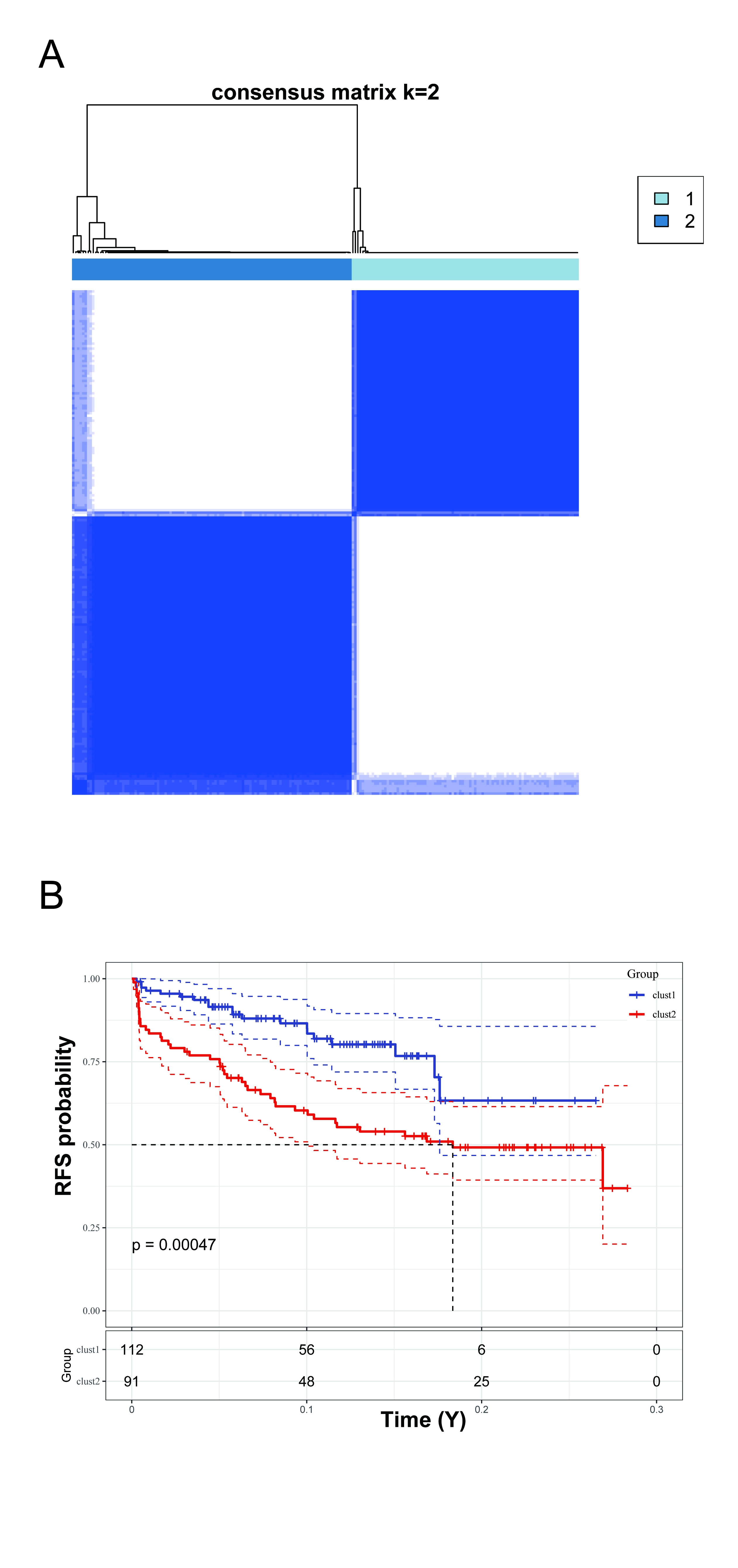

Supplement: Supplementary file 3 [file Image2.TIF]

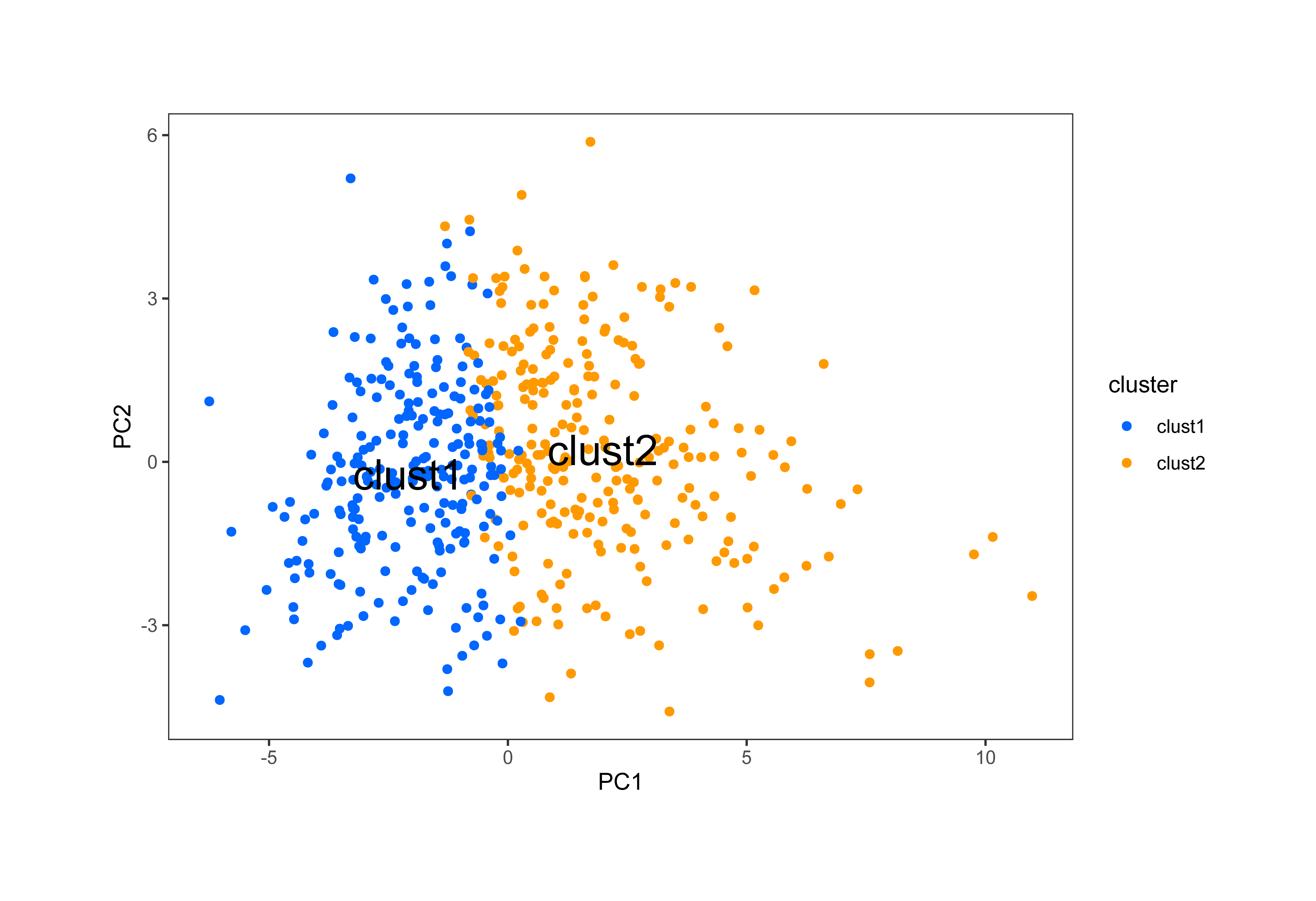

Supplement: Supplementary file 4 [file Image1.TIF]

A

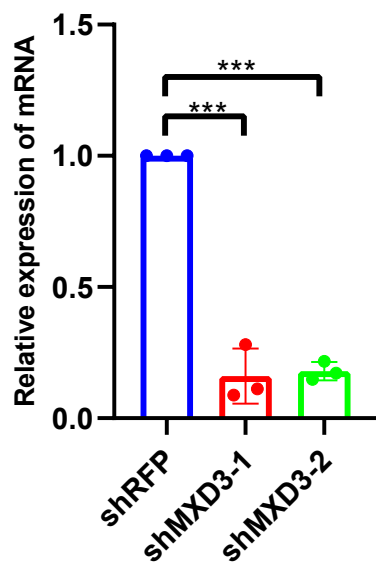

B

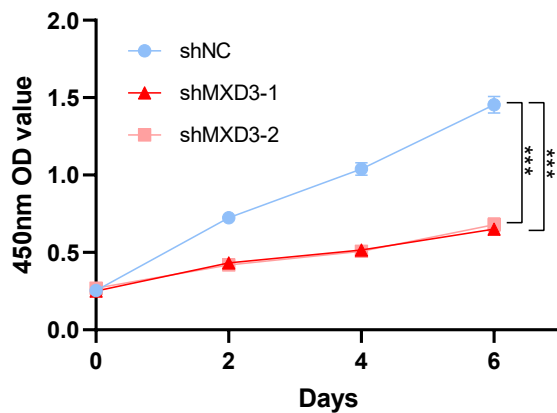

C

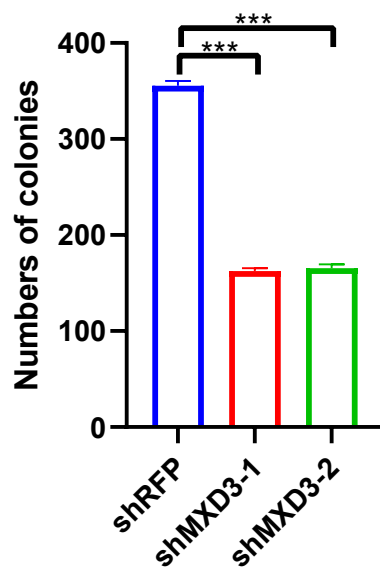

D

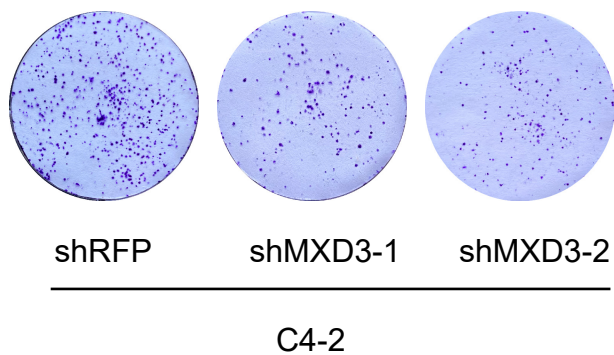

Supplement: Supplementary file 5 [file Image4.pdf]
