# Supplementary material for: Identification of molecular subtypes and a prognostic signature based on chromatin regulators related genes in prostate cancer
Source: Front Genet. 2023 Jan 10;13:1110723. doi: 10.3389/fgene.2022.1110723 (PMC9871366; doi:10.3389/fgene.2022.1110723)
Supplement: Supplementary file 7 [file DataSheet1.PDF]

| TCGA_coh | patient | status | time | Age | T stage | GS | PSA |
|---|---|---|---|---|---|---|---|
| 1 | TCGA_ZG_ | 0 | 132 | ⩾ 65 | T3–4 | ⩾ 8 | < 10 or unknown |
| 2 | TCGA_ZG_ | 0 | 410 | < 65 | T3–4 | ⩾ 8 | < 10 or unknown |
| 3 | TCGA_ZG_ | 0 | 349 | ⩾ 65 | T3–4 | ⩾ 8 | < 10 or unknown |
| 4 | TCGA_ZG_ | 0 | 455 | ⩾ 65 | T3–4 | ⩾ 8 | ⩾ 10 |
| 5 | TCGA_ZG_ | 0 | 547 | ⩾ 65 | T3–4 | ⩾ 8 | < 10 or unknown |
| 6 | TCGA_ZG_ | 0 | 692 | ⩾ 65 | T3–4 | ⩾ 8 | ⩾ 10 |
| 7 | TCGA_ZG_ | 0 | 370 | < 65 | T3–4 | ⩾ 8 | < 10 or unknown |
| 8 | TCGA_ZG_ | 0 | 579 | ⩾ 65 | T3–4 | ⩾ 8 | < 10 or unknown |
| 9 | TCGA_ZG_ | 0 | 516 | < 65 | T3–4 | ⩾ 8 | < 10 or unknown |
| 10 | TCGA_ZG_ | 0 | 115 | < 65 | T3–4 | ⩾ 8 | ⩾ 10 |
| 11 | TCGA_ZG_ | 0 | 378 | ⩾ 65 | T3–4 | ⩾ 8 | < 10 or unknown |
| 12 | TCGA_ZG_ | 0 | 583 | ⩾ 65 | T3–4 | ⩾ 8 | < 10 or unknown |
| 13 | TCGA_ZG_ | 1 | 51 | < 65 | T3–4 | ⩾ 8 | < 10 or unknown |
| 14 | TCGA_ZG_ | 1 | 664 | < 65 | T3–4 | ⩾ 8 | < 10 or unknown |
| 15 | TCGA_ZG_ | 0 | 908 | < 65 | T3–4 | ⩾ 8 | < 10 or unknown |
| 16 | TCGA_ZG_ | 0 | 1108 | < 65 | T3–4 | ⩾ 8 | < 10 or unknown |
| 17 | TCGA_ZG_ | 1 | 180 | ⩾ 65 | T3–4 | ⩾ 8 | < 10 or unknown |
| 18 | TCGA_ZG_ | 0 | 1302 | ⩾ 65 | T3–4 | ⩾ 8 | < 10 or unknown |
| 19 | TCGA_ZG_ | 0 | 355 | ⩾ 65 | T3–4 | ⩾ 8 | < 10 or unknown |
| 20 | TCGA_ZG_ | 0 | 130 | ⩾ 65 | T3–4 | ⩾ 8 | < 10 or unknown |
| 21 | TCGA_ZG_ | 0 | 308 | ⩾ 65 | T3–4 | ⩾ 8 | < 10 or unknown |
| 22 | TCGA_ZG_ | 0 | 404 | ⩾ 65 | T3–4 | ⩾ 8 | < 10 or unknown |
| 23 | TCGA_ZG_ | 0 | 442 | < 65 | T1–2 | < 8 | < 10 or unknown |
| 24 | TCGA_ZG_ | 0 | 94 | ⩾ 65 | T3–4 | ⩾ 8 | < 10 or unknown |
| 25 | TCGA_YL_ | 1 | 765 | < 65 | T3–4 | ⩾ 8 | < 10 or unknown |
| 26 | TCGA_YL_ | 1 | 1506 | ⩾ 65 | T3–4 | ⩾ 8 | < 10 or unknown |
| 27 | TCGA_YL_ | 1 | 740 | < 65 | T3–4 | ⩾ 8 | ⩾ 10 |
| 28 | TCGA_YL_ | 1 | 1009 | < 65 | T3–4 | ⩾ 8 | ⩾ 10 |
| 29 | TCGA_YL_ | 1 | 1476 | < 65 | T3–4 | ⩾ 8 | ⩾ 10 |
| 30 | TCGA_YL_ | 0 | 1623 | < 65 | T3–4 | ⩾ 8 | < 10 or unknown |
| 31 | TCGA_YL_ | 0 | 427 | ⩾ 65 | T3–4 | ⩾ 8 | < 10 or unknown |
| 32 | TCGA_YL_ | 0 | 273 | < 65 | T3–4 | ⩾ 8 | < 10 or unknown |
| 33 | TCGA_YL_ | 1 | 329 | < 65 | T3–4 | ⩾ 8 | < 10 or unknown |
| 34 | TCGA_YL_ | 1 | 2036 | < 65 | T3–4 | ⩾ 8 | < 10 or unknown |
| 35 | TCGA_YL_ | 0 | 3479 | < 65 | T3–4 | ⩾ 8 | < 10 or unknown |
| 36 | TCGA_YL_ | 0 | 987 | ⩾ 65 | T3–4 | ⩾ 8 | < 10 or unknown |
| 37 | TCGA_YL_ | 0 | 1408 | ⩾ 65 | T3–4 | ⩾ 8 | < 10 or unknown |
| 38 | TCGA_YL_ | 1 | 752 | < 65 | T3–4 | ⩾ 8 | < 10 or unknown |
| 39 | TCGA_YL_ | 1 | 1423 | ⩾ 65 | T3–4 | ⩾ 8 | < 10 or unknown |
| 40 | TCGA_YL_ | 0 | 875 | ⩾ 65 | T3–4 | < 8 | < 10 or unknown |
| 41 | TCGA_YL_ | 0 | 1373 | < 65 | T3–4 | ⩾ 8 | < 10 or unknown |
| 42 | TCGA_YL_ | 1 | 152 | ⩾ 65 | T3–4 | ⩾ 8 | < 10 or unknown |
| 43 | TCGA_YL_ | 1 | 1384 | < 65 | T3–4 | ⩾ 8 | < 10 or unknown |
| 44 | TCGA_YL_ | 0 | 512 | ⩾ 65 | T3–4 | ⩾ 8 | < 10 or unknown |
| 45 | TCGA_YL_ | 1 | 1006 | < 65 | T3–4 | ⩾ 8 | < 10 or unknown |
| 46 | TCGA_YL_ | 1 | 679 | ⩾ 65 | T3–4 | ⩾ 8 | < 10 or unknown |
| 47 | TCGA_YL_ | 1 | 1068 | ⩾ 65 | T3–4 | < 8 | < 10 or unknown |
| 48 | TCGA_YL_ | 1 | 1473 | ⩾ 65 | T3–4 | ⩾ 8 | < 10 or unknown |
| 49 | TCGA_YL_ | 0 | 1565 | < 65 | T3–4 | ⩾ 8 | < 10 or unknown |
| 50 | TCGA_YL_ | 1 | 1376 | < 65 | T3–4 | ⩾ 8 | < 10 or unknown |
| 51 | TCGA_YL_ | 0 | 1611 | < 65 | T3–4 | ⩾ 8 | < 10 or unknown |
| 52 | TCGA_YJ_ | 0 | 148 | ⩾ 65 | T3–4 | ⩾ 8 | < 10 or unknown |
| 54 | TCGA_Y6_ | 0 | 992 | ⩾ 65 | T3–4 | < 8 | < 10 or unknown |
| 55 | TCGA_XQ_ | 0 | 770 | ⩾ 65 | T3–4 | ⩾ 8 | < 10 or unknown |
| 57 | TCGA_XK_ | 0 | 979 | < 65 | T1–2 | < 8 | < 10 or unknown |
| 58 | TCGA_XK_ | 0 | 1576 | ⩾ 65 | T1–2 | < 8 | < 10 or unknown |
| 59 | TCGA_XK_ | 0 | 1434 | ⩾ 65 | T3–4 | < 8 | < 10 or unknown |

| | | | | | | | |
|---|---|---|---|---|---|---|---|
| 60 | TCGA_XK_ | 1 | 131 | < 65 | T3–4 | < 8 | < 10 or unknown |
| 61 | TCGA_XK_ | 0 | 1259 | ⩾ 65 | T3–4 | < 8 | < 10 or unknown |
| 62 | TCGA_XK_ | 0 | 930 | < 65 | T3–4 | < 8 | < 10 or unknown |
| 63 | TCGA_XK_ | 0 | 1262 | < 65 | T3–4 | ⩾ 8 | < 10 or unknown |
| 64 | TCGA_XK_ | 1 | 427 | ⩾ 65 | T3–4 | ⩾ 8 | < 10 or unknown |
| 65 | TCGA_XK_ | 0 | 1133 | < 65 | T3–4 | < 8 | < 10 or unknown |
| 66 | TCGA_XK_ | 0 | 972 | ⩾ 65 | T1–2 | ⩾ 8 | < 10 or unknown |
| 67 | TCGA_XJ_ | 0 | 973 | < 65 | T3–4 | ⩾ 8 | < 10 or unknown |
| 68 | TCGA_XJ_ | 0 | 91 | < 65 | T1–2 | < 8 | < 10 or unknown |
| 69 | TCGA_XJ_ | 0 | 275 | < 65 | T1–2 | ⩾ 8 | < 10 or unknown |
| 70 | TCGA_XJ_ | 0 | 1461 | < 65 | T3–4 | ⩾ 8 | < 10 or unknown |
| 71 | TCGA_XJ_ | 0 | 1280 | < 65 | T1–2 | < 8 | < 10 or unknown |
| 72 | TCGA_XJ_ | 0 | 1461 | < 65 | T3–4 | < 8 | < 10 or unknown |
| 73 | TCGA_XJ_ | 0 | 1127 | ⩾ 65 | T3–4 | < 8 | < 10 or unknown |
| 74 | TCGA_XA_ | 0 | 376 | ⩾ 65 | T1–2 | < 8 | < 10 or unknown |
| 75 | TCGA_X4_ | 0 | 979 | < 65 | T1–2 | < 8 | < 10 or unknown |
| 76 | TCGA_X4_ | 0 | 1383 | ⩾ 65 | T3–4 | ⩾ 8 | < 10 or unknown |
| 77 | TCGA_WW | 0 | 449 | ⩾ 65 | T1–2 | ⩾ 8 | < 10 or unknown |
| 78 | TCGA_VP_ | 0 | 1365 | ⩾ 65 | T3–4 | ⩾ 8 | < 10 or unknown |
| 79 | TCGA_VP_ | 1 | 533 | < 65 | T3–4 | ⩾ 8 | < 10 or unknown |
| 80 | TCGA_VP_ | 0 | 1774 | < 65 | T3–4 | < 8 | < 10 or unknown |
| 81 | TCGA_VP_ | 0 | 663 | ⩾ 65 | T3–4 | ⩾ 8 | < 10 or unknown |
| 82 | TCGA_VP_ | 0 | 2037 | < 65 | T1–2 | < 8 | < 10 or unknown |
| 83 | TCGA_VP_ | 1 | 1194 | < 65 | T3–4 | ⩾ 8 | < 10 or unknown |
| 84 | TCGA_VP_ | 0 | 1690 | ⩾ 65 | T1–2 | < 8 | < 10 or unknown |
| 85 | TCGA_VP_ | 1 | 2473 | < 65 | T1–2 | ⩾ 8 | ⩾ 10 |
| 86 | TCGA_VP_ | 0 | 728 | ⩾ 65 | T1–2 | ⩾ 8 | < 10 or unknown |
| 87 | TCGA_VP_ | 1 | 98 | < 65 | T1–2 | ⩾ 8 | < 10 or unknown |
| 88 | TCGA_VP_ | 0 | 3333 | < 65 | T1–2 | ⩾ 8 | < 10 or unknown |
| 89 | TCGA_VP_ | 0 | 2309 | ⩾ 65 | T3–4 | < 8 | < 10 or unknown |
| 90 | TCGA_VP_ | 0 | 3631 | < 65 | T3–4 | ⩾ 8 | < 10 or unknown |
| 91 | TCGA_VN_ | 0 | 496 | ⩾ 65 | T1–2 | ⩾ 8 | < 10 or unknown |
| 92 | TCGA_VN_ | 1 | 512 | < 65 | T1–2 | ⩾ 8 | < 10 or unknown |
| 93 | TCGA_VN_ | 0 | 1366 | < 65 | T1–2 | ⩾ 8 | < 10 or unknown |
| 94 | TCGA_VN_ | 0 | 1260 | < 65 | T1–2 | < 8 | < 10 or unknown |
| 95 | TCGA_VN_ | 0 | 481 | < 65 | T1–2 | < 8 | < 10 or unknown |
| 96 | TCGA_VN_ | 0 | 766 | < 65 | T1–2 | < 8 | < 10 or unknown |
| 97 | TCGA_VN_ | 0 | 189 | < 65 | T1–2 | < 8 | < 10 or unknown |
| 98 | TCGA_VN_ | 0 | 746 | < 65 | T1–2 | < 8 | < 10 or unknown |
| 99 | TCGA_VN_ | 0 | 776 | < 65 | T3–4 | ⩾ 8 | < 10 or unknown |
| 100 | TCGA_VN_ | 0 | 269 | < 65 | T3–4 | ⩾ 8 | < 10 or unknown |
| 101 | TCGA_V1_ | 0 | 1361 | ⩾ 65 | T3–4 | ⩾ 8 | < 10 or unknown |
| 102 | TCGA_V1_ | 0 | 1383 | ⩾ 65 | T3–4 | ⩾ 8 | < 10 or unknown |
| 103 | TCGA_V1_ | 0 | 1640 | ⩾ 65 | T3–4 | ⩾ 8 | ⩾ 10 |
| 104 | TCGA_V1_ | 0 | 1517 | < 65 | T1–2 | ⩾ 8 | < 10 or unknown |
| 105 | TCGA_V1_ | 0 | 540 | < 65 | T1–2 | ⩾ 8 | < 10 or unknown |
| 106 | TCGA_V1_ | 0 | 827 | < 65 | T3–4 | ⩾ 8 | < 10 or unknown |
| 107 | TCGA_V1_ | 0 | 874 | < 65 | T3–4 | ⩾ 8 | ⩾ 10 |
| 108 | TCGA_V1_ | 0 | 1067 | < 65 | T3–4 | < 8 | < 10 or unknown |
| 109 | TCGA_V1_ | 0 | 1134 | < 65 | T1–2 | ⩾ 8 | < 10 or unknown |
| 110 | TCGA_V1_ | 1 | 292 | < 65 | T1–2 | < 8 | < 10 or unknown |
| 111 | TCGA_V1_ | 0 | 371 | ⩾ 65 | T3–4 | < 8 | < 10 or unknown |
| 112 | TCGA_V1_ | 1 | 105 | ⩾ 65 | T3–4 | ⩾ 8 | < 10 or unknown |
| 113 | TCGA_V1_ | 0 | 2403 | < 65 | T1–2 | ⩾ 8 | < 10 or unknown |
| 114 | TCGA_V1_ | 0 | 1184 | < 65 | T1–2 | < 8 | < 10 or unknown |
| 115 | TCGA_V1_ | 0 | 636 | < 65 | T3–4 | ⩾ 8 | < 10 or unknown |
| 116 | TCGA_V1_ | 0 | 2870 | < 65 | T3–4 | ⩾ 8 | ⩾ 10 |
| 117 | TCGA_V1_ | 1 | 819 | < 65 | T3–4 | ⩾ 8 | ⩾ 10 |

| | | | | | | | |
|---|---|---|---|---|---|---|---|
| 118 | TCGA_V1_ | 1 | 124 | < 65 | T3–4 | ⩾ 8 | ⩾ 10 |
| 119 | TCGA_V1_ | 0 | 202 | < 65 | T1–2 | < 8 | < 10 or unknown |
| 120 | TCGA_V1_ | 0 | 1078 | < 65 | T3–4 | ⩾ 8 | < 10 or unknown |
| 121 | TCGA_V1_ | 0 | 686 | < 65 | T3–4 | ⩾ 8 | < 10 or unknown |
| 122 | TCGA_V1_ | 0 | 474 | < 65 | T3–4 | < 8 | < 10 or unknown |
| 123 | TCGA_V1_ | 0 | 924 | < 65 | T1–2 | < 8 | < 10 or unknown |
| 124 | TCGA_V1_ | 0 | 1948 | < 65 | T3–4 | < 8 | < 10 or unknown |
| 125 | TCGA_V1_ | 0 | 1876 | < 65 | T3–4 | < 8 | < 10 or unknown |
| 126 | TCGA_V1_ | 1 | 990 | < 65 | T3–4 | < 8 | < 10 or unknown |
| 127 | TCGA_V1_ | 0 | 448 | < 65 | T1–2 | < 8 | < 10 or unknown |
| 128 | TCGA_V1_ | 0 | 508 | < 65 | T1–2 | < 8 | < 10 or unknown |
| 129 | TCGA_V1_ | 0 | 1791 | < 65 | T3–4 | < 8 | < 10 or unknown |
| 130 | TCGA_V1_ | 0 | 857 | < 65 | T3–4 | < 8 | < 10 or unknown |
| 131 | TCGA_V1_ | 0 | 1449 | ⩾ 65 | T3–4 | < 8 | < 10 or unknown |
| 132 | TCGA_TP_ | 0 | 610 | < 65 | T1–2 | < 8 | < 10 or unknown |
| 133 | TCGA_TP_ | 0 | 526 | < 65 | T3–4 | < 8 | < 10 or unknown |
| 135 | TCGA_SU_ | 0 | 551 | < 65 | T1–2 | ⩾ 8 | < 10 or unknown |
| 136 | TCGA_QU_ | 0 | 2620 | ⩾ 65 | T1–2 | < 8 | < 10 or unknown |
| 137 | TCGA_QU_ | 0 | 3716 | < 65 | T1–2 | < 8 | < 10 or unknown |
| 138 | TCGA_QU_ | 0 | 4264 | < 65 | T1–2 | < 8 | < 10 or unknown |
| 139 | TCGA_QU_ | 0 | 1247 | < 65 | T1–2 | < 8 | < 10 or unknown |
| 140 | TCGA_QU_ | 0 | 97 | < 65 | T1–2 | < 8 | < 10 or unknown |
| 141 | TCGA_MG_ | 0 | 173 | < 65 | T3–4 | ⩾ 8 | < 10 or unknown |
| 142 | TCGA_M7_ | 0 | 573 | < 65 | T3–4 | < 8 | < 10 or unknown |
| 143 | TCGA_M7_ | 0 | 889 | < 65 | T3–4 | ⩾ 8 | < 10 or unknown |
| 144 | TCGA_M7_ | 0 | 763 | < 65 | T3–4 | < 8 | < 10 or unknown |
| 145 | TCGA_M7_ | 1 | 559 | ⩾ 65 | T3–4 | ⩾ 8 | < 10 or unknown |
| 146 | TCGA_M7_ | 0 | 400 | ⩾ 65 | T1–2 | < 8 | < 10 or unknown |
| 147 | TCGA_M7_ | 0 | 384 | < 65 | T1–2 | < 8 | < 10 or unknown |
| 148 | TCGA_M7_ | 0 | 643 | < 65 | T3–4 | < 8 | < 10 or unknown |
| 149 | TCGA_M7_ | 0 | 493 | < 65 | T1–2 | < 8 | < 10 or unknown |
| 150 | TCGA_KK_ | 0 | 1915 | < 65 | T1–2 | < 8 | < 10 or unknown |
| 151 | TCGA_KK_ | 0 | 628 | ⩾ 65 | T3–4 | ⩾ 8 | < 10 or unknown |
| 152 | TCGA_KK_ | 0 | 2052 | < 65 | T1–2 | ⩾ 8 | < 10 or unknown |
| 153 | TCGA_KK_ | 1 | 307 | < 65 | T3–4 | < 8 | < 10 or unknown |
| 154 | TCGA_KK_ | 1 | 626 | < 65 | T3–4 | ⩾ 8 | ⩾ 10 |
| 155 | TCGA_KK_ | 0 | 2013 | < 65 | T3–4 | < 8 | < 10 or unknown |
| 156 | TCGA_KK_ | 0 | 2505 | < 65 | T3–4 | < 8 | < 10 or unknown |
| 157 | TCGA_KK_ | 1 | 648 | < 65 | T3–4 | < 8 | < 10 or unknown |
| 158 | TCGA_KK_ | 0 | 2107 | ⩾ 65 | T3–4 | ⩾ 8 | < 10 or unknown |
| 159 | TCGA_KK_ | 1 | 1060 | < 65 | T3–4 | ⩾ 8 | < 10 or unknown |
| 160 | TCGA_KK_ | 0 | 83 | ⩾ 65 | T3–4 | ⩾ 8 | < 10 or unknown |
| 161 | TCGA_KK_ | 0 | 1931 | ⩾ 65 | T3–4 | ⩾ 8 | < 10 or unknown |
| 162 | TCGA_KK_ | 1 | 940 | < 65 | T3–4 | ⩾ 8 | < 10 or unknown |
| 163 | TCGA_KK_ | 0 | 967 | ⩾ 65 | T3–4 | ⩾ 8 | < 10 or unknown |
| 164 | TCGA_KK_ | 1 | 1088 | < 65 | T3–4 | ⩾ 8 | < 10 or unknown |
| 165 | TCGA_KK_ | 0 | 668 | < 65 | T3–4 | < 8 | < 10 or unknown |
| 166 | TCGA_KK_ | 0 | 3096 | < 65 | T1–2 | < 8 | < 10 or unknown |
| 167 | TCGA_KK_ | 1 | 1162 | < 65 | T3–4 | < 8 | < 10 or unknown |
| 168 | TCGA_KK_ | 1 | 637 | < 65 | T3–4 | ⩾ 8 | < 10 or unknown |
| 169 | TCGA_KK_ | 1 | 294 | < 65 | T3–4 | ⩾ 8 | < 10 or unknown |
| 170 | TCGA_KK_ | 1 | 692 | ⩾ 65 | T3–4 | ⩾ 8 | < 10 or unknown |
| 171 | TCGA_KK_ | 0 | 1063 | ⩾ 65 | T3–4 | < 8 | < 10 or unknown |
| 172 | TCGA_KK_ | 1 | 606 | ⩾ 65 | T3–4 | ⩾ 8 | < 10 or unknown |
| 173 | TCGA_KK_ | 0 | 1519 | < 65 | T3–4 | < 8 | < 10 or unknown |
| 174 | TCGA_KK_ | 1 | 1124 | < 65 | T1–2 | < 8 | < 10 or unknown |
| 175 | TCGA_KK_ | 0 | 1054 | < 65 | T3–4 | < 8 | < 10 or unknown |
| 176 | TCGA_KK_ | 0 | 822 | < 65 | T1–2 | < 8 | < 10 or unknown |

| | | | | | | | |
|---|---|---|---|---|---|---|---|
| 177 | TCGA_KK_A | 1 | 207 | < 65 | T3–4 | ⩾ 8 | < 10 or unknown |
| 178 | TCGA_KK_A | 1 | 1217 | < 65 | T1–2 | < 8 | < 10 or unknown |
| 179 | TCGA_KK_A | 0 | 196 | < 65 | T3–4 | ⩾ 8 | < 10 or unknown |
| 180 | TCGA_KK_A | 0 | 1770 | ⩾ 65 | T1–2 | ⩾ 8 | < 10 or unknown |
| 181 | TCGA_KK_A | 1 | 925 | < 65 | T3–4 | ⩾ 8 | < 10 or unknown |
| 182 | TCGA_KK_A | 0 | 3447 | ⩾ 65 | T3–4 | ⩾ 8 | < 10 or unknown |
| 183 | TCGA_KK_A | 0 | 2073 | < 65 | T3–4 | < 8 | < 10 or unknown |
| 184 | TCGA_KK_A | 0 | 3502 | ⩾ 65 | T1–2 | < 8 | < 10 or unknown |
| 185 | TCGA_KK_A | 0 | 2056 | < 65 | T1–2 | < 8 | < 10 or unknown |
| 186 | TCGA_KK_A | 0 | 5024 | < 65 | T1–2 | < 8 | < 10 or unknown |
| 187 | TCGA_KK_A | 0 | 2198 | < 65 | T3–4 | ⩾ 8 | < 10 or unknown |
| 188 | TCGA_KK_A | 1 | 941 | < 65 | T3–4 | ⩾ 8 | < 10 or unknown |
| 189 | TCGA_KK_A | 0 | 4295 | < 65 | T1–2 | < 8 | < 10 or unknown |
| 190 | TCGA_KK_A | 1 | 203 | ⩾ 65 | T3–4 | ⩾ 8 | < 10 or unknown |
| 191 | TCGA_KK_A | 0 | 2628 | ⩾ 65 | T1–2 | < 8 | < 10 or unknown |
| 192 | TCGA_KK_A | 0 | 1968 | < 65 | T3–4 | ⩾ 8 | < 10 or unknown |
| 193 | TCGA_KK_A | 1 | 2233 | < 65 | T3–4 | ⩾ 8 | < 10 or unknown |
| 194 | TCGA_KK_A | 0 | 3440 | < 65 | T3–4 | ⩾ 8 | < 10 or unknown |
| 195 | TCGA_KC_A | 0 | 352 | < 65 | T1–2 | < 8 | < 10 or unknown |
| 196 | TCGA_KC_A | 0 | 270 | < 65 | T3–4 | < 8 | < 10 or unknown |
| 197 | TCGA_KC_A | 0 | 598 | < 65 | T3–4 | < 8 | < 10 or unknown |
| 198 | TCGA_KC_A | 0 | 263 | < 65 | T1–2 | < 8 | < 10 or unknown |
| 199 | TCGA_KC_A | 0 | 91 | < 65 | T1–2 | < 8 | < 10 or unknown |
| 200 | TCGA_KC_A | 0 | 665 | ⩾ 65 | T1–2 | < 8 | < 10 or unknown |
| 201 | TCGA_KC_A | 1 | 1328 | ⩾ 65 | T3–4 | ⩾ 8 | < 10 or unknown |
| 202 | TCGA_KC_A | 1 | 1022 | ⩾ 65 | T3–4 | ⩾ 8 | < 10 or unknown |
| 203 | TCGA_KC_A | 0 | 607 | < 65 | T1–2 | < 8 | < 10 or unknown |
| 204 | TCGA_KC_A | 0 | 1815 | < 65 | T1–2 | < 8 | < 10 or unknown |
| 205 | TCGA_KC_A | 1 | 193 | ⩾ 65 | T1–2 | < 8 | ⩾ 10 |
| 206 | TCGA_J9_A | 0 | 386 | ⩾ 65 | T3–4 | < 8 | < 10 or unknown |
| 207 | TCGA_J9_A | 0 | 1237 | < 65 | T3–4 | < 8 | < 10 or unknown |
| 208 | TCGA_J9_A | 1 | 344 | ⩾ 65 | T3–4 | ⩾ 8 | < 10 or unknown |
| 209 | TCGA_J9_A | 1 | 132 | ⩾ 65 | T3–4 | ⩾ 8 | ⩾ 10 |
| 210 | TCGA_J9_A | 0 | 321 | ⩾ 65 | T3–4 | ⩾ 8 | < 10 or unknown |
| 211 | TCGA_J9_A | 0 | 323 | ⩾ 65 | T3–4 | ⩾ 8 | < 10 or unknown |
| 212 | TCGA_J9_A | 0 | 212 | ⩾ 65 | T3–4 | ⩾ 8 | < 10 or unknown |
| 213 | TCGA_J9_A | 0 | 178 | < 65 | T3–4 | ⩾ 8 | < 10 or unknown |
| 214 | TCGA_J9_A | 1 | 57 | < 65 | T3–4 | ⩾ 8 | < 10 or unknown |
| 215 | TCGA_J4_A | 0 | 825 | < 65 | T1–2 | < 8 | < 10 or unknown |
| 216 | TCGA_J4_A | 1 | 79 | ⩾ 65 | T3–4 | ⩾ 8 | < 10 or unknown |
| 217 | TCGA_J4_A | 0 | 553 | ⩾ 65 | T1–2 | < 8 | < 10 or unknown |
| 218 | TCGA_J4_A | 1 | 433 | < 65 | T1–2 | < 8 | < 10 or unknown |
| 219 | TCGA_J4_A | 1 | 521 | < 65 | T1–2 | < 8 | < 10 or unknown |
| 220 | TCGA_J4_A | 0 | 720 | < 65 | T1–2 | < 8 | < 10 or unknown |
| 221 | TCGA_J4_A | 0 | 328 | < 65 | T1–2 | < 8 | < 10 or unknown |
| 222 | TCGA_J4_A | 0 | 696 | ⩾ 65 | T1–2 | < 8 | < 10 or unknown |
| 223 | TCGA_J4_A | 0 | 685 | < 65 | T3–4 | < 8 | < 10 or unknown |
| 224 | TCGA_J4_A | 0 | 512 | < 65 | T1–2 | < 8 | < 10 or unknown |
| 225 | TCGA_J4_A | 1 | 618 | < 65 | T3–4 | ⩾ 8 | < 10 or unknown |
| 226 | TCGA_J4_A | 0 | 769 | ⩾ 65 | T3–4 | ⩾ 8 | < 10 or unknown |
| 227 | TCGA_J4_A | 0 | 183 | < 65 | T1–2 | < 8 | < 10 or unknown |
| 228 | TCGA_J4_A | 1 | 708 | < 65 | T3–4 | < 8 | < 10 or unknown |
| 229 | TCGA_J4_A | 0 | 910 | ⩾ 65 | T1–2 | < 8 | < 10 or unknown |
| 230 | TCGA_J4_A | 0 | 975 | ⩾ 65 | T1–2 | < 8 | < 10 or unknown |
| 231 | TCGA_J4_A | 0 | 839 | < 65 | T1–2 | < 8 | < 10 or unknown |
| 232 | TCGA_J4_A | 1 | 442 | < 65 | T3–4 | < 8 | < 10 or unknown |
| 233 | TCGA_J4_A | 0 | 941 | < 65 | T3–4 | < 8 | < 10 or unknown |
| 234 | TCGA_J4_A | 0 | 770 | < 65 | T3–4 | < 8 | < 10 or unknown |

| | | | | | | | |
|---|---|---|---|---|---|---|---|
| 235 | TCGA_J4_A | 0 | 1004 | ≥ 65 | T1–2 | < 8 | < 10 or unknown |
| 236 | TCGA_J4_8 | 0 | 1218 | < 65 | T1–2 | < 8 | < 10 or unknown |
| 237 | TCGA_J4_8 | 0 | 614 | < 65 | T3–4 | < 8 | < 10 or unknown |
| 238 | TCGA_HI_7 | 1 | 1329 | < 65 | T3–4 | ≥ 8 | < 10 or unknown |
| 239 | TCGA_HI_7 | 0 | 2522 | < 65 | T1–2 | < 8 | < 10 or unknown |
| 240 | TCGA_HI_7 | 0 | 2684 | < 65 | T3–4 | < 8 | < 10 or unknown |
| 241 | TCGA_HI_7 | 1 | 2505 | < 65 | T3–4 | ≥ 8 | < 10 or unknown |
| 242 | TCGA_HC_ | 1 | 351 | < 65 | T3–4 | ≥ 8 | < 10 or unknown |
| 243 | TCGA_HC_ | 1 | 216 | < 65 | T3–4 | ≥ 8 | < 10 or unknown |
| 244 | TCGA_HC_ | 0 | 573 | ≥ 65 | T1–2 | < 8 | < 10 or unknown |
| 245 | TCGA_HC_ | 0 | 775 | < 65 | T1–2 | < 8 | < 10 or unknown |
| 246 | TCGA_HC_ | 0 | 290 | < 65 | T3–4 | ≥ 8 | < 10 or unknown |
| 247 | TCGA_HC_ | 0 | 150 | < 65 | T1–2 | < 8 | < 10 or unknown |
| 248 | TCGA_HC_ | 0 | 213 | ≥ 65 | T3–4 | < 8 | < 10 or unknown |
| 249 | TCGA_HC_ | 0 | 134 | < 65 | T1–2 | < 8 | < 10 or unknown |
| 250 | TCGA_HC_ | 0 | 38 | < 65 | T1–2 | < 8 | < 10 or unknown |
| 251 | TCGA_HC_ | 0 | 44 | ≥ 65 | T1–2 | < 8 | < 10 or unknown |
| 252 | TCGA_HC_ | 0 | 106 | < 65 | T1–2 | < 8 | < 10 or unknown |
| 253 | TCGA_HC_ | 0 | 71 | < 65 | T1–2 | < 8 | < 10 or unknown |
| 254 | TCGA_HC_ | 0 | 530 | < 65 | T1–2 | < 8 | < 10 or unknown |
| 255 | TCGA_HC_ | 0 | 49 | ≥ 65 | T3–4 | < 8 | < 10 or unknown |
| 256 | TCGA_HC_ | 0 | 67 | < 65 | T1–2 | < 8 | < 10 or unknown |
| 257 | TCGA_HC_ | 0 | 61 | ≥ 65 | T3–4 | ≥ 8 | < 10 or unknown |
| 258 | TCGA_HC_ | 0 | 54 | ≥ 65 | T1–2 | ≥ 8 | < 10 or unknown |
| 259 | TCGA_HC_ | 0 | 23 | < 65 | T3–4 | ≥ 8 | < 10 or unknown |
| 260 | TCGA_HC_ | 0 | 680 | < 65 | T1–2 | ≥ 8 | < 10 or unknown |
| 261 | TCGA_HC_ | 0 | 473 | < 65 | T3–4 | ≥ 8 | < 10 or unknown |
| 262 | TCGA_HC_ | 0 | 483 | ≥ 65 | T3–4 | ≥ 8 | < 10 or unknown |
| 263 | TCGA_HC_ | 0 | 48 | < 65 | T3–4 | ≥ 8 | < 10 or unknown |
| 264 | TCGA_HC_ | 0 | 679 | < 65 | T1–2 | ≥ 8 | < 10 or unknown |
| 265 | TCGA_HC_ | 0 | 546 | < 65 | T1–2 | < 8 | < 10 or unknown |
| 266 | TCGA_HC_ | 0 | 686 | < 65 | T1–2 | < 8 | < 10 or unknown |
| 267 | TCGA_HC_ | 0 | 924 | < 65 | T1–2 | < 8 | < 10 or unknown |
| 268 | TCGA_HC_ | 0 | 847 | < 65 | T1–2 | < 8 | < 10 or unknown |
| 269 | TCGA_HC_ | 0 | 726 | ≥ 65 | T3–4 | < 8 | < 10 or unknown |
| 270 | TCGA_HC_ | 0 | 860 | < 65 | T1–2 | < 8 | < 10 or unknown |
| 271 | TCGA_HC_ | 0 | 682 | < 65 | T3–4 | < 8 | < 10 or unknown |
| 272 | TCGA_HC_ | 0 | 589 | < 65 | T1–2 | < 8 | < 10 or unknown |
| 273 | TCGA_HC_ | 0 | 956 | < 65 | T3–4 | ≥ 8 | < 10 or unknown |
| 274 | TCGA_HC_ | 0 | 747 | ≥ 65 | T1–2 | < 8 | < 10 or unknown |
| 275 | TCGA_HC_ | 0 | 543 | < 65 | T1–2 | ≥ 8 | < 10 or unknown |
| 276 | TCGA_HC_ | 0 | 938 | ≥ 65 | T1–2 | < 8 | < 10 or unknown |
| 277 | TCGA_HC_ | 0 | 928 | < 65 | T3–4 | < 8 | < 10 or unknown |
| 278 | TCGA_HC_ | 0 | 782 | < 65 | T1–2 | < 8 | < 10 or unknown |
| 279 | TCGA_HC_ | 0 | 522 | < 65 | T1–2 | < 8 | < 10 or unknown |
| 280 | TCGA_HC_ | 0 | 724 | ≥ 65 | T3–4 | < 8 | < 10 or unknown |
| 281 | TCGA_HC_ | 0 | 792 | < 65 | T1–2 | < 8 | < 10 or unknown |
| 282 | TCGA_HC_ | 0 | 748 | < 65 | T1–2 | < 8 | < 10 or unknown |
| 283 | TCGA_HC_ | 0 | 721 | ≥ 65 | T3–4 | < 8 | < 10 or unknown |
| 284 | TCGA_HC_ | 0 | 531 | < 65 | T3–4 | < 8 | < 10 or unknown |
| 285 | TCGA_HC_ | 0 | 938 | < 65 | T3–4 | < 8 | < 10 or unknown |
| 286 | TCGA_HC_ | 0 | 717 | < 65 | T1–2 | < 8 | < 10 or unknown |
| 287 | TCGA_HC_ | 1 | 420 | < 65 | T1–2 | < 8 | < 10 or unknown |
| 288 | TCGA_HC_ | 0 | 1090 | < 65 | T1–2 | < 8 | < 10 or unknown |
| 289 | TCGA_HC_ | 0 | 1103 | < 65 | T1–2 | < 8 | < 10 or unknown |
| 290 | TCGA_HC_ | 0 | 1355 | ≥ 65 | T1–2 | < 8 | < 10 or unknown |
| 291 | TCGA_HC_ | 1 | 766 | ≥ 65 | T3–4 | ≥ 8 | < 10 or unknown |
| 292 | TCGA_HC_ | 0 | 939 | ≥ 65 | T1–2 | < 8 | < 10 or unknown |

| | | | | | | | |
|---|---|---|---|---|---|---|---|
| 293 | TCGA_HC_ | 0 | 1001 | < 65 | T1–2 | < 8 | ⩾ 10 |
| 294 | TCGA_HC_ | 1 | 170 | < 65 | T3–4 | ⩾ 8 | ⩾ 10 |
| 295 | TCGA_HC_ | 0 | 870 | < 65 | T1–2 | < 8 | < 10 or unknown |
| 296 | TCGA_HC_ | 0 | 1221 | < 65 | T1–2 | < 8 | ⩾ 10 |
| 297 | TCGA_HC_ | 0 | 868 | ⩾ 65 | T1–2 | < 8 | < 10 or unknown |
| 298 | TCGA_HC_ | 0 | 440 | < 65 | T1–2 | < 8 | < 10 or unknown |
| 299 | TCGA_HC_ | 0 | 1136 | < 65 | T3–4 | ⩾ 8 | < 10 or unknown |
| 300 | TCGA_HC_ | 1 | 894 | ⩾ 65 | T1–2 | < 8 | < 10 or unknown |
| 301 | TCGA_HC_ | 1 | 380 | < 65 | T3–4 | < 8 | < 10 or unknown |
| 302 | TCGA_HC_ | 0 | 1985 | ⩾ 65 | T3–4 | < 8 | < 10 or unknown |
| 303 | TCGA_HC_ | 0 | 2115 | < 65 | T3–4 | < 8 | < 10 or unknown |
| 304 | TCGA_HC_ | 0 | 601 | < 65 | T1–2 | < 8 | < 10 or unknown |
| 305 | TCGA_H9_ | 0 | 112 | < 65 | T3–4 | < 8 | < 10 or unknown |
| 306 | TCGA_H9_ | 0 | 941 | < 65 | T1–2 | < 8 | < 10 or unknown |
| 307 | TCGA_H9_ | 0 | 185 | ⩾ 65 | T1–2 | < 8 | < 10 or unknown |
| 308 | TCGA_G9_ | 0 | 728 | < 65 | T3–4 | ⩾ 8 | < 10 or unknown |
| 309 | TCGA_G9_ | 0 | 906 | < 65 | T3–4 | ⩾ 8 | < 10 or unknown |
| 310 | TCGA_G9_ | 1 | 423 | < 65 | T3–4 | ⩾ 8 | ⩾ 10 |
| 311 | TCGA_G9_ | 0 | 994 | < 65 | T1–2 | < 8 | < 10 or unknown |
| 312 | TCGA_G9_ | 0 | 857 | < 65 | T3–4 | < 8 | < 10 or unknown |
| 313 | TCGA_G9_ | 0 | 1078 | < 65 | T1–2 | < 8 | < 10 or unknown |
| 314 | TCGA_G9_ | 0 | 942 | < 65 | T3–4 | ⩾ 8 | < 10 or unknown |
| 315 | TCGA_G9_ | 0 | 849 | < 65 | T1–2 | < 8 | < 10 or unknown |
| 316 | TCGA_G9_ | 0 | 1185 | ⩾ 65 | T3–4 | ⩾ 8 | < 10 or unknown |
| 317 | TCGA_G9_ | 0 | 1610 | < 65 | T1–2 | < 8 | < 10 or unknown |
| 318 | TCGA_G9_ | 0 | 1543 | ⩾ 65 | T3–4 | ⩾ 8 | < 10 or unknown |
| 319 | TCGA_G9_ | 1 | 1342 | < 65 | T3–4 | < 8 | < 10 or unknown |
| 320 | TCGA_G9_ | 0 | 1726 | < 65 | T1–2 | < 8 | < 10 or unknown |
| 321 | TCGA_G9_ | 0 | 1771 | ⩾ 65 | T3–4 | < 8 | < 10 or unknown |
| 322 | TCGA_G9_ | 0 | 830 | ⩾ 65 | T1–2 | < 8 | < 10 or unknown |
| 323 | TCGA_G9_ | 0 | 765 | < 65 | T3–4 | < 8 | < 10 or unknown |
| 324 | TCGA_G9_ | 0 | 1778 | ⩾ 65 | T3–4 | < 8 | < 10 or unknown |
| 325 | TCGA_G9_ | 0 | 1155 | < 65 | T1–2 | < 8 | < 10 or unknown |
| 326 | TCGA_G9_ | 0 | 958 | < 65 | T1–2 | < 8 | < 10 or unknown |
| 327 | TCGA_G9_ | 0 | 811 | ⩾ 65 | T3–4 | < 8 | < 10 or unknown |
| 328 | TCGA_G9_ | 0 | 1226 | < 65 | T1–2 | < 8 | < 10 or unknown |
| 329 | TCGA_G9_ | 0 | 1156 | < 65 | T3–4 | < 8 | < 10 or unknown |
| 330 | TCGA_G9_ | 0 | 1215 | < 65 | T1–2 | < 8 | < 10 or unknown |
| 331 | TCGA_G9_ | 0 | 1222 | < 65 | T3–4 | ⩾ 8 | < 10 or unknown |
| 332 | TCGA_G9_ | 0 | 1947 | < 65 | T3–4 | < 8 | < 10 or unknown |
| 333 | TCGA_G9_ | 0 | 1363 | ⩾ 65 | T3–4 | < 8 | < 10 or unknown |
| 334 | TCGA_G9_ | 0 | 1198 | ⩾ 65 | T3–4 | < 8 | < 10 or unknown |
| 335 | TCGA_G9_ | 0 | 1378 | < 65 | T3–4 | < 8 | < 10 or unknown |
| 336 | TCGA_G9_ | 0 | 1443 | < 65 | T3–4 | < 8 | < 10 or unknown |
| 337 | TCGA_G9_ | 0 | 1415 | < 65 | T3–4 | < 8 | < 10 or unknown |
| 338 | TCGA_G9_ | 0 | 1434 | < 65 | T3–4 | ⩾ 8 | < 10 or unknown |
| 339 | TCGA_G9_ | 0 | 2066 | < 65 | T1–2 | < 8 | < 10 or unknown |
| 340 | TCGA_G9_ | 0 | 1542 | < 65 | T3–4 | < 8 | < 10 or unknown |
| 341 | TCGA_G9_ | 0 | 2048 | < 65 | T1–2 | < 8 | < 10 or unknown |
| 342 | TCGA_G9_ | 0 | 1515 | ⩾ 65 | T1–2 | < 8 | < 10 or unknown |
| 343 | TCGA_G9_ | 0 | 2089 | < 65 | T1–2 | < 8 | < 10 or unknown |
| 344 | TCGA_G9_ | 0 | 2450 | < 65 | T1–2 | < 8 | < 10 or unknown |
| 345 | TCGA_G9_ | 0 | 1696 | < 65 | T3–4 | < 8 | < 10 or unknown |
| 346 | TCGA_G9_ | 1 | 1634 | < 65 | T3–4 | < 8 | < 10 or unknown |
| 347 | TCGA_G9_ | 0 | 2028 | < 65 | T3–4 | < 8 | < 10 or unknown |
| 349 | TCGA_G9_ | 0 | 2465 | ⩾ 65 | T1–2 | < 8 | < 10 or unknown |
| 350 | TCGA_G9_ | 1 | 1180 | < 65 | T3–4 | < 8 | < 10 or unknown |
| 351 | TCGA_G9_ | 0 | 1266 | ⩾ 65 | T1–2 | < 8 | < 10 or unknown |

| | | | | | | | |
|---|---|---|---|---|---|---|---|
| 352 | TCGA_FC_/ | 0 | 616 | ⩾ 65 | T3–4 | < 8 | ⩾ 10 |
| 353 | TCGA_FC_/ | 0 | 789 | ⩾ 65 | T3–4 | < 8 | < 10 or unknown |
| 354 | TCGA_FC_/ | 0 | 524 | ⩾ 65 | T3–4 | < 8 | ⩾ 10 |
| 355 | TCGA_FC_/ | 0 | 680 | < 65 | T3–4 | ⩾ 8 | ⩾ 10 |
| 356 | TCGA_FC_/ | 0 | 877 | ⩾ 65 | T3–4 | ⩾ 8 | < 10 or unknown |
| 357 | TCGA_FC_ | 0 | 469 | < 65 | T3–4 | ⩾ 8 | < 10 or unknown |
| 358 | TCGA_FC_ | 0 | 864 | < 65 | T3–4 | < 8 | < 10 or unknown |
| 359 | TCGA_EJ_A | 0 | 145 | < 65 | T1–2 | < 8 | < 10 or unknown |
| 360 | TCGA_EJ_A | 0 | 131 | < 65 | T1–2 | < 8 | < 10 or unknown |
| 361 | TCGA_EJ_A | 0 | 138 | < 65 | T3–4 | ⩾ 8 | < 10 or unknown |
| 362 | TCGA_EJ_A | 1 | 216 | ⩾ 65 | T3–4 | < 8 | < 10 or unknown |
| 363 | TCGA_EJ_A | 1 | 117 | < 65 | T1–2 | ⩾ 8 | < 10 or unknown |
| 364 | TCGA_EJ_A | 0 | 282 | < 65 | T3–4 | < 8 | < 10 or unknown |
| 365 | TCGA_EJ_A | 0 | 329 | < 65 | T1–2 | < 8 | < 10 or unknown |
| 366 | TCGA_EJ_A | 1 | 197 | < 65 | T3–4 | < 8 | < 10 or unknown |
| 367 | TCGA_EJ_A | 0 | 128 | < 65 | T3–4 | ⩾ 8 | < 10 or unknown |
| 368 | TCGA_EJ_A | 0 | 476 | < 65 | T3–4 | < 8 | < 10 or unknown |
| 369 | TCGA_EJ_A | 0 | 197 | < 65 | T1–2 | ⩾ 8 | < 10 or unknown |
| 370 | TCGA_EJ_A | 0 | 474 | < 65 | T1–2 | < 8 | < 10 or unknown |
| 371 | TCGA_EJ_A | 0 | 432 | < 65 | T3–4 | < 8 | < 10 or unknown |
| 372 | TCGA_EJ_A | 0 | 250 | < 65 | T1–2 | < 8 | < 10 or unknown |
| 374 | TCGA_EJ_A | 1 | 353 | ⩾ 65 | T3–4 | ⩾ 8 | < 10 or unknown |
| 375 | TCGA_EJ_A | 0 | 230 | ⩾ 65 | T1–2 | < 8 | < 10 or unknown |
| 376 | TCGA_EJ_A | 0 | 451 | < 65 | T3–4 | ⩾ 8 | < 10 or unknown |
| 377 | TCGA_EJ_A | 0 | 666 | < 65 | T1–2 | ⩾ 8 | < 10 or unknown |
| 378 | TCGA_EJ_A | 1 | 75 | < 65 | T3–4 | ⩾ 8 | < 10 or unknown |
| 379 | TCGA_EJ_A | 0 | 417 | ⩾ 65 | T3–4 | < 8 | < 10 or unknown |
| 380 | TCGA_EJ_A | 0 | 393 | ⩾ 65 | T3–4 | ⩾ 8 | < 10 or unknown |
| 381 | TCGA_EJ_A | 0 | 710 | < 65 | T3–4 | ⩾ 8 | < 10 or unknown |
| 382 | TCGA_EJ_A | 0 | 668 | < 65 | T3–4 | < 8 | < 10 or unknown |
| 383 | TCGA_EJ_A | 0 | 724 | < 65 | T1–2 | < 8 | < 10 or unknown |
| 384 | TCGA_EJ_A | 0 | 668 | ⩾ 65 | T3–4 | ⩾ 8 | < 10 or unknown |
| 385 | TCGA_EJ_A | 1 | 215 | < 65 | T3–4 | ⩾ 8 | ⩾ 10 |
| 387 | TCGA_EJ_A | 0 | 626 | < 65 | T1–2 | ⩾ 8 | < 10 or unknown |
| 388 | TCGA_EJ_A | 0 | 657 | ⩾ 65 | T3–4 | ⩾ 8 | < 10 or unknown |
| 389 | TCGA_EJ_8 | 0 | 766 | ⩾ 65 | T3–4 | ⩾ 8 | < 10 or unknown |
| 390 | TCGA_EJ_8 | 1 | 196 | < 65 | T3–4 | ⩾ 8 | < 10 or unknown |
| 391 | TCGA_EJ_8 | 0 | 1159 | < 65 | T1–2 | < 8 | < 10 or unknown |
| 392 | TCGA_EJ_8 | 1 | 1925 | < 65 | T3–4 | ⩾ 8 | ⩾ 10 |
| 393 | TCGA_EJ_8 | 0 | 2347 | < 65 | T3–4 | ⩾ 8 | < 10 or unknown |
| 394 | TCGA_EJ_7 | 0 | 983 | < 65 | T1–2 | < 8 | < 10 or unknown |
| 395 | TCGA_EJ_7 | 0 | 1306 | ⩾ 65 | T1–2 | < 8 | < 10 or unknown |
| 396 | TCGA_EJ_7 | 0 | 114 | < 65 | T1–2 | < 8 | < 10 or unknown |
| 397 | TCGA_EJ_7 | 0 | 1396 | < 65 | T1–2 | < 8 | < 10 or unknown |
| 398 | TCGA_EJ_7 | 0 | 1118 | ⩾ 65 | T1–2 | < 8 | < 10 or unknown |
| 399 | TCGA_EJ_7 | 0 | 1105 | ⩾ 65 | T3–4 | < 8 | < 10 or unknown |
| 400 | TCGA_EJ_7 | 0 | 859 | < 65 | T3–4 | < 8 | < 10 or unknown |
| 401 | TCGA_EJ_7 | 0 | 1096 | < 65 | T1–2 | < 8 | < 10 or unknown |
| 402 | TCGA_EJ_7 | 0 | 1177 | < 65 | T3–4 | < 8 | < 10 or unknown |
| 403 | TCGA_EJ_7 | 0 | 781 | < 65 | T1–2 | < 8 | < 10 or unknown |
| 404 | TCGA_EJ_7 | 0 | 787 | ⩾ 65 | T3–4 | < 8 | < 10 or unknown |
| 405 | TCGA_EJ_7 | 0 | 1167 | ⩾ 65 | T1–2 | ⩾ 8 | < 10 or unknown |
| 406 | TCGA_EJ_7 | 0 | 1073 | ⩾ 65 | T1–2 | < 8 | < 10 or unknown |
| 407 | TCGA_EJ_7 | 0 | 742 | < 65 | T1–2 | < 8 | < 10 or unknown |
| 408 | TCGA_EJ_7 | 0 | 191 | ⩾ 65 | T3–4 | < 8 | < 10 or unknown |
| 409 | TCGA_EJ_7 | 0 | 842 | ⩾ 65 | T3–4 | < 8 | < 10 or unknown |
| 410 | TCGA_EJ_7 | 0 | 923 | < 65 | T3–4 | < 8 | < 10 or unknown |
| 411 | TCGA_EJ_7 | 0 | 1070 | < 65 | T3–4 | < 8 | < 10 or unknown |

| | | | | | | | |
|---|---|---|---|---|---|---|---|
| 412 | TCGA_EJ_7 | 0 | 824 | < 65 | T3–4 | < 8 | < 10 or unknown |
| 413 | TCGA_EJ_7 | 1 | 380 | < 65 | T3–4 | < 8 | < 10 or unknown |
| 414 | TCGA_EJ_7 | 0 | 771 | ⩾ 65 | T1–2 | < 8 | < 10 or unknown |
| 415 | TCGA_EJ_7 | 0 | 1008 | ⩾ 65 | T3–4 | < 8 | < 10 or unknown |
| 416 | TCGA_EJ_7 | 0 | 1150 | < 65 | T3–4 | < 8 | < 10 or unknown |
| 417 | TCGA_EJ_7 | 0 | 1099 | < 65 | T3–4 | < 8 | < 10 or unknown |
| 418 | TCGA_EJ_7 | 0 | 2542 | ⩾ 65 | T1–2 | < 8 | < 10 or unknown |
| 419 | TCGA_EJ_7 | 0 | 2850 | < 65 | T1–2 | < 8 | < 10 or unknown |
| 420 | TCGA_EJ_7 | 0 | 2572 | < 65 | T1–2 | < 8 | < 10 or unknown |
| 421 | TCGA_EJ_7 | 0 | 2687 | ⩾ 65 | T3–4 | < 8 | < 10 or unknown |
| 422 | TCGA_EJ_5 | 0 | 1513 | < 65 | T3–4 | < 8 | < 10 or unknown |
| 423 | TCGA_EJ_5 | 0 | 1828 | < 65 | T3–4 | < 8 | < 10 or unknown |
| 424 | TCGA_EJ_5 | 0 | 1273 | < 65 | T3–4 | < 8 | < 10 or unknown |
| 425 | TCGA_EJ_5 | 0 | 1832 | < 65 | T1–2 | < 8 | < 10 or unknown |
| 426 | TCGA_EJ_5 | 0 | 1778 | ⩾ 65 | T3–4 | < 8 | < 10 or unknown |
| 427 | TCGA_EJ_5 | 1 | 537 | < 65 | T3–4 | ⩾ 8 | < 10 or unknown |
| 428 | TCGA_EJ_5 | 1 | 546 | ⩾ 65 | T1–2 | ⩾ 8 | ⩾ 10 |
| 429 | TCGA_EJ_5 | 1 | 265 | < 65 | T3–4 | ⩾ 8 | < 10 or unknown |
| 430 | TCGA_EJ_5 | 0 | 2079 | < 65 | T1–2 | < 8 | < 10 or unknown |
| 431 | TCGA_EJ_5 | 0 | 2279 | < 65 | T3–4 | < 8 | < 10 or unknown |
| 432 | TCGA_EJ_5 | 0 | 1962 | < 65 | T3–4 | ⩾ 8 | < 10 or unknown |
| 433 | TCGA_EJ_5 | 1 | 2104 | ⩾ 65 | T3–4 | ⩾ 8 | < 10 or unknown |
| 434 | TCGA_EJ_5 | 0 | 1889 | < 65 | T1–2 | < 8 | < 10 or unknown |
| 435 | TCGA_EJ_5 | 0 | 1889 | < 65 | T3–4 | < 8 | < 10 or unknown |
| 436 | TCGA_EJ_5 | 0 | 1832 | < 65 | T3–4 | < 8 | < 10 or unknown |
| 437 | TCGA_EJ_5 | 0 | 1829 | ⩾ 65 | T1–2 | ⩾ 8 | < 10 or unknown |
| 438 | TCGA_EJ_5 | 0 | 1733 | < 65 | T1–2 | < 8 | < 10 or unknown |
| 439 | TCGA_EJ_5 | 0 | 1476 | < 65 | T3–4 | < 8 | < 10 or unknown |
| 440 | TCGA_EJ_5 | 0 | 1887 | < 65 | T1–2 | < 8 | < 10 or unknown |
| 441 | TCGA_EJ_5 | 0 | 1464 | < 65 | T1–2 | < 8 | < 10 or unknown |
| 442 | TCGA_EJ_5 | 0 | 1965 | ⩾ 65 | T3–4 | < 8 | < 10 or unknown |
| 443 | TCGA_EJ_5 | 0 | 1457 | < 65 | T3–4 | ⩾ 8 | < 10 or unknown |
| 444 | TCGA_EJ_5 | 0 | 1381 | ⩾ 65 | T3–4 | ⩾ 8 | < 10 or unknown |
| 445 | TCGA_EJ_5 | 0 | 922 | < 65 | T1–2 | < 8 | < 10 or unknown |
| 446 | TCGA_EJ_5 | 0 | 1427 | ⩾ 65 | T3–4 | < 8 | < 10 or unknown |
| 447 | TCGA_EJ_5 | 0 | 1391 | < 65 | T3–4 | ⩾ 8 | < 10 or unknown |
| 448 | TCGA_EJ_5 | 0 | 1171 | < 65 | T1–2 | < 8 | < 10 or unknown |
| 449 | TCGA_EJ_5 | 0 | 1365 | < 65 | T3–4 | < 8 | < 10 or unknown |
| 450 | TCGA_EJ_5 | 0 | 1415 | < 65 | T3–4 | < 8 | < 10 or unknown |
| 451 | TCGA_EJ_5 | 0 | 1720 | < 65 | T1–2 | < 8 | < 10 or unknown |
| 452 | TCGA_EJ_5 | 0 | 405 | < 65 | T1–2 | < 8 | < 10 or unknown |
| 453 | TCGA_EJ_5 | 0 | 595 | < 65 | T1–2 | < 8 | < 10 or unknown |
| 454 | TCGA_EJ_5 | 0 | 1760 | ⩾ 65 | T3–4 | ⩾ 8 | < 10 or unknown |
| 455 | TCGA_EJ_5 | 0 | 1477 | < 65 | T3–4 | < 8 | < 10 or unknown |
| 456 | TCGA_CH_ | 0 | 882 | ⩾ 65 | T1–2 | < 8 | < 10 or unknown |
| 457 | TCGA_CH_ | 0 | 91 | < 65 | T3–4 | ⩾ 8 | ⩾ 10 |
| 458 | TCGA_CH_ | 1 | 396 | ⩾ 65 | T3–4 | < 8 | < 10 or unknown |
| 459 | TCGA_CH_ | 0 | 974 | < 65 | T1–2 | < 8 | < 10 or unknown |
| 460 | TCGA_CH_ | 0 | 304 | < 65 | T3–4 | < 8 | < 10 or unknown |
| 461 | TCGA_CH_ | 0 | 821 | ⩾ 65 | T3–4 | < 8 | ⩾ 10 |
| 462 | TCGA_CH_ | 0 | 486 | < 65 | T3–4 | ⩾ 8 | < 10 or unknown |
| 463 | TCGA_CH_ | 0 | 396 | < 65 | T1–2 | < 8 | < 10 or unknown |
| 464 | TCGA_CH_ | 0 | 62 | < 65 | T3–4 | ⩾ 8 | ⩾ 10 |
| 465 | TCGA_CH_ | 0 | 731 | ⩾ 65 | T3–4 | < 8 | < 10 or unknown |
| 466 | TCGA_CH_ | 0 | 458 | ⩾ 65 | T1–2 | < 8 | < 10 or unknown |
| 467 | TCGA_CH_ | 0 | 31 | < 65 | T3–4 | < 8 | ⩾ 10 |
| 468 | TCGA_CH_ | 0 | 700 | < 65 | T3–4 | < 8 | < 10 or unknown |
| 469 | TCGA_CH_ | 0 | 31 | ⩾ 65 | T3–4 | < 8 | ⩾ 10 |

| | | | | | | | |
|---|---|---|---|---|---|---|---|
| 470 | TCGA_CH_ | 0 | 365 | ≥ 65 | T3–4 | < 8 | < 10 or unknown |
| 471 | TCGA_CH_ | 0 | 1339 | < 65 | T3–4 | < 8 | < 10 or unknown |
| 472 | TCGA_CH_ | 0 | 28 | < 65 | T3–4 | ≥ 8 | ≥ 10 |
| 473 | TCGA_CH_ | 0 | 62 | ≥ 65 | T3–4 | ≥ 8 | ≥ 10 |
| 474 | TCGA_CH_ | 0 | 31 | ≥ 65 | T3–4 | ≥ 8 | ≥ 10 |
| 475 | TCGA_CH_ | 0 | 943 | ≥ 65 | T3–4 | ≥ 8 | < 10 or unknown |
| 476 | TCGA_CH_ | 1 | 365 | ≥ 65 | T3–4 | < 8 | < 10 or unknown |
| 477 | TCGA_CH_ | 0 | 396 | ≥ 65 | T1–2 | < 8 | < 10 or unknown |
| 478 | TCGA_CH_ | 0 | 31 | < 65 | T3–4 | < 8 | < 10 or unknown |
| 479 | TCGA_CH_ | 0 | 731 | < 65 | T1–2 | < 8 | < 10 or unknown |
| 480 | TCGA_CH_ | 0 | 91 | ≥ 65 | T3–4 | < 8 | ≥ 10 |
| 481 | TCGA_CH_ | 0 | 60 | < 65 | T1–2 | < 8 | ≥ 10 |
| 482 | TCGA_CH_ | 1 | 425 | ≥ 65 | T1–2 | < 8 | < 10 or unknown |
| 483 | TCGA_CH_ | 0 | 395 | < 65 | T3–4 | ≥ 8 | < 10 or unknown |
| 484 | TCGA_CH_ | 0 | 31 | < 65 | T1–2 | < 8 | < 10 or unknown |
| 485 | TCGA_CH_ | 0 | 671 | ≥ 65 | T3–4 | < 8 | < 10 or unknown |
| 487 | TCGA_CH_ | 0 | 91 | ≥ 65 | T1–2 | < 8 | ≥ 10 |
| 488 | TCGA_4L_ | 0 | 350 | < 65 | T3–4 | ≥ 8 | ≥ 10 |
| 489 | TCGA_2A_ | 0 | 615 | < 65 | T3–4 | < 8 | < 10 or unknown |
| 490 | TCGA_2A_ | 0 | 1272 | < 65 | T1–2 | < 8 | < 10 or unknown |
| 491 | TCGA_2A_ | 0 | 1364 | < 65 | T3–4 | < 8 | < 10 or unknown |
| 492 | TCGA_2A_ | 1 | 198 | ≥ 65 | T3–4 | ≥ 8 | < 10 or unknown |
| 493 | TCGA_2A_ | 0 | 112 | < 65 | T3–4 | < 8 | < 10 or unknown |
| 494 | TCGA_2A_ | 0 | 1378 | ≥ 65 | T3–4 | ≥ 8 | < 10 or unknown |
| 495 | TCGA_2A_ | 0 | 671 | < 65 | T1–2 | < 8 | < 10 or unknown |
| 496 | TCGA_2A_ | 0 | 1373 | < 65 | T3–4 | ≥ 8 | < 10 or unknown |
| 497 | TCGA_2A_ | 0 | 1701 | < 65 | T3–4 | < 8 | < 10 or unknown |
| 498 | TCGA_2A_ | 0 | 621 | < 65 | T1–2 | < 8 | < 10 or unknown |

| Name | RFSstatus | RFStime | Age | Gleason | PSA | Tstage | SURGICAL MARGINS |
|---|---|---|---|---|---|---|---|
| GSM18177 | 0 | 65.42466 | 69 | 7 | 8.3 | T3 | POSITIVE |
| GSM18177 | 0 | 64.01096 | 69 | 7 | 15.3 | T3 | POSITIVE |
| GSM18177 | 0 | 64.47123 | 57 | 7 | 4.8 | T2 | NEGATIVE |
| GSM18177 | 1 | 6 | 58 | 7 | 4.3 | T2 | NEGATIVE |
| GSM18177 | 0 | 48.52603 | 62 | 7 | 5.3 | T3 | NEGATIVE |
| GSM18177 | 1 | 1 | 55 | 9 | 9.8 | T4 | POSITIVE |
| GSM18177 | 0 | 61.51233 | 65 | 7 | 5.8 | T3 | NEGATIVE |
| GSM18177 | 1 | 55 | 55 | 7 | 13.9 | T3 | NEGATIVE |
| GSM18177 | 0 | 54.11507 | 57 | 7 | 6 | T3 | NEGATIVE |
| GSM18177 | 0 | 41.91781 | 57 | 6 | 7.8 | T2 | NEGATIVE |
| GSM18177 | 0 | 25.9726 | 51 | 6 | 8.8 | T2 | NEGATIVE |
| GSM18177 | 0 | 59.63836 | 62 | 6 | 6.5 | T2 | NEGATIVE |
| GSM18177 | 0 | 59.47397 | 61 | 6 | 14.5 | T3 | POSITIVE |
| GSM18177 | 0 | 59.90137 | 63 | 7 | 9.8 | T3 | NEGATIVE |
| GSM18177 | 0 | 44.87671 | 63 | 7 | 4.9 | T3 | NEGATIVE |
| GSM18177 | 0 | 52.86575 | 63 | 7 | 7.5 | T2 | NEGATIVE |
| GSM18177 | 0 | 48.88767 | 61 | 6 | 8.7 | T2 | NEGATIVE |
| GSM18177 | 0 | 58.22466 | 59 | 6 | 9.3 | T2 | NEGATIVE |
| GSM18177 | 0 | 58.5863 | 67 | 6 | 3.2 | T3 | POSITIVE |
| GSM18177 | 0 | 43.46301 | 42 | 7 | 11.8 | T2 | NEGATIVE |
| GSM18177 | 0 | 51.35342 | 64 | 6 | 12 | T3 | NEGATIVE |
| GSM18177 | 0 | 57.36986 | 54 | 7 | 12 | T3 | NEGATIVE |
| GSM18177 | 0 | 46.84932 | 62 | 7 | 7 | T3 | POSITIVE |
| GSM18177 | 0 | 23.47397 | 63 | 7 | 8 | T3 | POSITIVE |
| GSM18177 | 1 | 11 | 59 | 8 | 5 | T3 | NEGATIVE |
| GSM18177 | 0 | 57.36986 | 61 | 7 | 3.6 | T3 | NEGATIVE |
| GSM18177 | 1 | 57 | 71 | 7 | 7.9 | T3 | NEGATIVE |
| GSM18177 | 0 | 57.36986 | 67 | 7 | 5.8 | T3 | POSITIVE |
| GSM18177 | 1 | 38 | 64 | 7 | 7.3 | T3 | POSITIVE |
| GSM18177 | 0 | 56.97534 | 72 | 6 | UNKNOWI | T2c | NEGATIVE |
| GSM18177 | 0 | 41.7863 | 61 | 7 | 6 | T3 | POSITIVE |
| GSM18177 | 0 | 54 | 62 | 7 | 17.3 | T3 | NEGATIVE |
| GSM18177 | 1 | 16 | 66 | 7 | 9.6 | T3 | NEGATIVE |
| GSM18177 | 0 | 47.24384 | 64 | 7 | 23.7 | T3 | NEGATIVE |
| GSM18177 | 0 | 52.70137 | 48 | 7 | 5.3 | T3 | POSITIVE |
| GSM18177 | 0 | 53.88493 | 63 | 7 | 9.2 | T3 | NEGATIVE |
| GSM18177 | 0 | 16.8 | 41 | 7 | 16.2 | T3 | NEGATIVE |
| GSM18177 | 0 | 52.27397 | 70 | 7 | 4.68 | T2 | NEGATIVE |
| GSM18177 | 0 | 51.78082 | 60 | 7 | 5.1 | T2 | NEGATIVE |
| GSM18177 | 0 | 50.69589 | 47 | 8 | 11.5 | T3 | NEGATIVE |
| GSM18177 | 0 | 50.86027 | 67 | 7 | 11 | T3 | NEGATIVE |
| GSM18177 | 0 | 50.23562 | 67 | 7 | 8.9 | T2 | NEGATIVE |
| GSM18177 | 0 | 48.95342 | 64 | 6 | 8.7 | T2 | NEGATIVE |
| GSM18177 | 0 | 33.99452 | 62 | 7 | 11.2 | T3 | NEGATIVE |
| GSM18177 | 0 | 48.46027 | 65 | 7 | 6.2 | T2 | NEGATIVE |
| GSM18177 | 0 | 38.59726 | 54 | 6 | 7.1 | T3 | NEGATIVE |
| GSM18177 | 0 | 47.24384 | 51 | 7 | 5.8 | T2 | NEGATIVE |
| GSM18177 | 0 | 46.0274 | 56 | 7 | 5.3 | T3 | NEGATIVE |
| GSM18177 | 1 | 13 | 69 | 7 | 6.2 | T3 | POSITIVE |
| GSM18177 | 1 | 21 | 60 | 8 | 7.9 | T3 | NEGATIVE |
| GSM18177 | 0 | 44.25205 | 53 | 7 | 5.7 | T2 | NEGATIVE |
| GSM18177 | 0 | 45.50137 | 67 | 6 | 13 | T2 | POSITIVE |
| GSM18177 | 1 | 31 | 55 | 6 | 4.6 | T3 | NEGATIVE |
| GSM18177 | 0 | 40.73425 | 63 | 6 | 8.5 | T3 | NEGATIVE |
| GSM18177 | 0 | 41.45753 | 69 | 7 | 15.8 | T3 | NEGATIVE |
| GSM18177 | 0 | 40.8 | 67 | 6 | 4.5 | T3 | NEGATIVE |
| GSM18177 | 1 | 16 | 54 | 7 | 5 | T3 | NEGATIVE |

| | | | | | | | |
|---|---|---|---|---|---|---|---|
| GSM18177 | 0 | 38.13699 | 56 | 7 | 9.7 | T3 | NEGATIVE |
| GSM18177 | 0 | 35.90137 | 50 | 7 | 11.9 | T3 | POSITIVE |
| GSM18177 | 0 | 22.55342 | 63 | 7 | 11.4 | T3 | POSITIVE |
| GSM18177 | 0 | 34.48767 | 56 | 7 | 7 | T2 | NEGATIVE |
| GSM18177 | 1 | 6 | 66 | 7 | 9 | T2 | POSITIVE |
| GSM18177 | 0 | 27.97808 | 69 | 7 | 7.5 | T3 | NEGATIVE |
| GSM18177 | 0 | 33.89589 | 72 | 7 | 9 | T2 | NEGATIVE |
| GSM18177 | 0 | 32.12055 | 59 | 7 | 4.2 | T3 | NEGATIVE |
| GSM18177 | 0 | 30.80548 | 63 | 7 | 7.55 | T3 | NEGATIVE |
| GSM18177 | 0 | 30.93699 | 58 | 7 | 6.6 | T2 | NEGATIVE |
| GSM18177 | 0 | 29.75342 | 62 | 7 | 16.8 | T3 | NEGATIVE |
| GSM18177 | 1 | 2 | 55 | 7 | 6.07 | T3 | POSITIVE |
| GSM18177 | 0 | 29.58904 | 48 | 7 | 6.92 | T3 | NEGATIVE |
| GSM18177 | 0 | 30.7726 | 57 | 7 | 8.5 | T3 | NEGATIVE |
| GSM18177 | 1 | 23 | 68 | 7 | 11.5 | T3 | NEGATIVE |
| GSM18177 | 1 | 2 | 67 | 7 | 7 | T3 | NEGATIVE |
| GSM18177 | 1 | 3 | 64 | 8 | 8.6 | T2 | NEGATIVE |
| GSM18177 | 0 | 27.02466 | 56 | 7 | 6.1 | T2 | POSITIVE |
| GSM18177 | 0 | 27.02466 | 44 | 7 | 4.3 | T3 | NEGATIVE |
| GSM18177 | 0 | 15.28767 | 50 | 6 | 8.3 | T2 | NEGATIVE |
| GSM18177 | 0 | 25.77534 | 73 | 7 | 5.2 | T2 | NEGATIVE |
| GSM18177 | 0 | 22.22466 | 60 | 8 | 10 | T3 | NEGATIVE |
| GSM18178 | 0 | 26.33425 | 62 | 7 | 12.08 | T3 | NEGATIVE |
| GSM18178 | 0 | 16.99726 | 52 | 7 | 13 | T2 | POSITIVE |
| GSM18178 | 0 | 15.28767 | 73 | 7 | 7.2 | T2 | NEGATIVE |
| GSM18178 | 0 | 22.32329 | 70 | 7 | 17.4 | T2 | NEGATIVE |
| GSM18178 | 0 | 15.45205 | 66 | 8 | 10 | T3 | NEGATIVE |
| GSM18178 | 0 | 22.29041 | 52 | 7 | 4 | T3 | NEGATIVE |
| GSM18178 | 0 | 15.22192 | 65 | 7 | 5 | T2 | NEGATIVE |
| GSM18178 | 0 | 21.73151 | 64 | 6 | 7.57 | T3 | NEGATIVE |
| GSM18178 | 0 | 21.20548 | 58 | 7 | 4.67 | T3 | NEGATIVE |
| GSM18178 | 0 | 20.71233 | 60 | 7 | 6.8 | T2 | POSITIVE |
| GSM18178 | 1 | 21 | 62 | 7 | 5.4 | T3 | NEGATIVE |
| GSM18178 | 0 | 21.27123 | 55 | 7 | 6.46 | T3 | NEGATIVE |
| GSM18178 | 0 | 20.02192 | 59 | 7 | 6.3 | T3 | NEGATIVE |
| GSM18178 | 0 | 18.14795 | 66 | 7 | 9.21 | T3 | POSITIVE |
| GSM18178 | 0 | 3.912329 | 60 | 7 | 10.95 | T3 | POSITIVE |
| GSM18178 | 1 | 1 | 57 | 7 | 6.8 | T3 | POSITIVE |
| GSM18178 | 0 | 18.83836 | 54 | 7 | 7.4 | T3 | NEGATIVE |
| GSM18178 | 0 | 18.44384 | 62 | 7 | 11.13 | T3 | NEGATIVE |
| GSM18178 | 0 | 7.857534 | 55 | 7 | 7.19 | T3 | NEGATIVE |
| GSM18178 | 0 | 19.46301 | 61 | 7 | 8.8 | T3 | NEGATIVE |
| GSM18178 | 1 | 19 | 61 | 7 | 11 | T3 | NEGATIVE |
| GSM18178 | 0 | 17.3589 | 63 | 8 | 9.61 | T3 | POSITIVE |
| GSM18178 | 0 | 9.928767 | 65 | 7 | 5 | T3 | NEGATIVE |
| GSM18178 | 0 | 15.25479 | 62 | 8 | 6.87 | T3 | NEGATIVE |
| GSM18178 | 0 | 10.55342 | 65 | 7 | 4.6 | T3 | POSITIVE |
| GSM18178 | 0 | 17.42466 | 54 | 7 | 13.6 | T3 | NEGATIVE |
| GSM18178 | 0 | 15.22192 | 61 | 7 | 7.63 | T3 | NEGATIVE |
| GSM18178 | 0 | 14.36712 | 67 | 7 | 7.8 | T3 | NEGATIVE |
| GSM18178 | 0 | 11.53973 | 56 | 7 | 13.89 | T2 | POSITIVE |
| GSM18178 | 0 | 10.5863 | 62 | 7 | 18 | T3 | NEGATIVE |
| GSM18178 | 0 | 12.69041 | 50 | 7 | 8.05 | T2 | NEGATIVE |
| GSM18178 | 0 | 10.45479 | 58 | 7 | 9.08 | T2 | NEGATIVE |
| GSM18179 | 1 | 12.03288 | | 7 | 15 | T3b | NEGATIVE |
| GSM18179 | 1 | 19.29863 | | 7 | 17 | T2a | POSITIVE |
| GSM18179 | 1 | 0.361644 | | 7 | 24 | T3a | POSITIVE |
| GSM18179 | 1 | 61.54521 | | 7 | 3.4 | T3a | NEGATIVE |

| | | | | | | |
|---|---|---|---|---|---|---|
| GSM18179 | 1 | 10.12603 | 7 | 11 | T3a | POSITIVE |
| GSM18179 | 1 | 8.087671 | 7 | 10 | T3b | POSITIVE |
| GSM18179 | 1 | 24.16438 | 9 | UNKNOWI | T3a | NEGATIVE |
| GSM18179 | 1 | 7.627397 | 9 | 117 | TxM | NEGATIVE |
| GSM18179 | 1 | 3.583562 | 9 | 6.6 | T3b | POSITIVE |
| GSM18179 | 1 | 2.926027 | 9 | 8.1 | T3b | POSITIVE |
| GSM18179 | 0 | 61.44658 | 7 | 4.8 | T2b | NEGATIVE |
| GSM18179 | 1 | 18.31233 | 6 | 3.2 | T3a | POSITIVE |
| GSM18179 | 1 | 1.380822 | 8 | 42 | T3b | POSITIVE |
| GSM18179 | 1 | 98.26849 | 7 | 7.3 | T3b | NEGATIVE |
| GSM18179 | 0 | 18.93699 | 7 | 6.6 | T3a | NEGATIVE |
| GSM18179 | 1 | 34.06027 | 7 | 5.1 | T2b | POSITIVE |
| GSM18179 | 1 | 1.578082 | 9 | 6.3 | T3b | POSITIVE |
| GSM18179 | 0 | 103.4301 | 8 | 6.4 | T3a | POSITIVE |
| GSM18179 | 0 | 100.274 | 6 | UNKNOWI | T2b | POSITIVE |
| GSM18179 | 1 | 1.413699 | 7 | 6.8 | T3a | POSITIVE |
| GSM18179 | 0 | 98.10411 | 5 | 7.4 | T2c | POSITIVE |
| GSM18179 | 0 | 57.0411 | 6 | 8.5 | T2M | NEGATIVE |
| GSM18179 | 1 | 26.86027 | 7 | 22 | T2b | POSITIVE |
| GSM18179 | 1 | 28.93151 | 7 | 7.8 | T3b | NEGATIVE |
| GSM18179 | 1 | 16.33973 | 9 | 6.4 | T3a | NEGATIVE |
| GSM18179 | 0 | 90.73973 | 6 | 6.6 | T2M | NEGATIVE |
| GSM18179 | 0 | 52.73425 | 7 | 12.7 | T2b | NEGATIVE |
| GSM18179 | 0 | 92.15342 | 6 | 13 | T2a | POSITIVE |
| GSM18179 | 0 | 77.62192 | 7 | 15 | T3a | POSITIVE |
| GSM18179 | 0 | 96.85479 | 7 | 8.2 | T3 | NEGATIVE |
| GSM18179 | 0 | 47.14521 | 6 | 9 | T2b | POSITIVE |
| GSM18179 | 1 | 63.15616 | 7 | 13 | T3b | POSITIVE |
| GSM18179 | 0 | 82.42192 | 6 | 6 | T2b | POSITIVE |
| GSM18179 | 0 | 1.808219 | 6 | 6.5 | T2b | NEGATIVE |
| GSM18179 | 1 | 1.808219 | 9 | 15.4 | T3a | POSITIVE |
| GSM18179 | 0 | 66 | 7 | 6 | T3 | NEGATIVE |
| GSM18179 | 0 | 87.3863 | 7 | 9.4 | T2c | NEGATIVE |
| GSM18179 | 1 | 23.70411 | 7 | 3.5 | T2b | NEGATIVE |
| GSM18179 | 0 | 98.33425 | 7 | 10 | T3a | NEGATIVE |
| GSM18179 | 0 | 95.90137 | 6 | 4.4 | T2b | NEGATIVE |
| GSM18179 | 0 | 94.5863 | 7 | 9.9 | T2b | POSITIVE |
| GSM18179 | 1 | 41.72055 | 7 | 20 | T2b | NEGATIVE |
| GSM18179 | 1 | 1.775342 | 10 | 9.8 | T3b | POSITIVE |
| GSM18179 | 0 | 82.55342 | 6 | 5.1 | T3a | POSITIVE |
| GSM18179 | 0 | 92.48219 | 7 | 7.6 | T2b | POSITIVE |
| GSM18179 | 0 | 62.33425 | 6 | 10 | T2c | POSITIVE |
| GSM18179 | 0 | 83.83562 | 5 | 9.2 | T2b | NEGATIVE |
| GSM18179 | 0 | 84.29589 | 6 | 16 | T2b | NEGATIVE |
| GSM18179 | 1 | 30.01644 | 8 | 4.3 | T2b | NEGATIVE |
| GSM18179 | 1 | 36.5589 | 7 | 20 | T3a | POSITIVE |
| GSM18179 | 1 | 22.98082 | 7 | 7.5 | T3a | NEGATIVE |
| GSM18179 | 0 | 84.19726 | 7 | 10 | T3b | POSITIVE |
| GSM18179 | 0 | 46.98082 | unknown | 3.8 | T2c | NEGATIVE |
| GSM18179 | 0 | 82.68493 | unknown | 11 | TxM | NEGATIVE |
| GSM18179 | 1 | 66.96986 | 7 | 11.2 | T2b | NEGATIVE |
| GSM18179 | 0 | 76.70137 | 7 | 7.2 | T2b | POSITIVE |
| GSM18179 | 0 | 91.59452 | 7 | 6.2 | T2b | NEGATIVE |
| GSM18179 | 1 | 19.89041 | 9 | 5.3 | T3a | NEGATIVE |
| GSM18179 | 0 | 78.7726 | 6 | 10 | T2b | NEGATIVE |
| GSM18179 | 0 | 77.26027 | 7 | 5.4 | T3a | NEGATIVE |
| GSM18179 | 1 | 36.59178 | 7 | 1.5 | T3a | NEGATIVE |
| GSM18179 | 0 | 84.06575 | 6 | 6.6 | T2b | NEGATIVE |

| | | | | | | |
|---|---|---|---|---|---|---|
| GSM18179 | 0 | 82.48767 | 6 | 12 | T2b | NEGATIVE |
| GSM18179 | 0 | 84.29589 | 6 | 5.1 | T2a | NEGATIVE |
| GSM18179 | 1 | 36.69041 | 8 | 7.5 | T3b | POSITIVE |
| GSM18179 | 0 | 85.54521 | 6 | 4.2 | T2b | NEGATIVE |
| GSM18179 | 0 | 61.18356 | 9 | 32 | T2b | NEGATIVE |
| GSM18179 | 0 | 79.79178 | 7 | 5.6 | T2b | NEGATIVE |
| GSM18179 | 1 | 0.854795 | 8 | 2.2 | T3a | POSITIVE |
| GSM18179 | 1 | 47.60548 | 7 | 14 | T3a | POSITIVE |
| GSM18179 | 1 | 1.413699 | 7 | 10 | T3a | NEGATIVE |
| GSM18179 | 0 | 79.43014 | 7 | 5.4 | T2b | NEGATIVE |
| GSM18179 | 0 | 78.04932 | 7 | 15 | T3a | POSITIVE |
| GSM18179 | 1 | 42.47671 | 7 | 11 | T2b | POSITIVE |
| GSM18179 | 1 | 42.80548 | 7 | 4.5 | T2b | POSITIVE |
| GSM18179 | 1 | 6.246575 | 7 | 17 | T2b | NEGATIVE |
| GSM18179 | 1 | 1.512329 | 7 | 22 | T3b | POSITIVE |
| GSM18179 | 0 | 74.33425 | 7 | 8.7 | T2b | POSITIVE |
| GSM18179 | 1 | 1.019178 | 7 | 8.2 | T2c | NEGATIVE |
| GSM18179 | 0 | 70.12603 | 6 | 7 | T0M | NEGATIVE |
| GSM18179 | 1 | 18.44384 | 7 | 3.7 | T2b | NEGATIVE |
| GSM18179 | 0 | 68.54795 | 7 | 4 | T2b | NEGATIVE |
| GSM18179 | 1 | 64.2411 | 7 | 4.7 | T3a | POSITIVE |
| GSM18179 | 1 | 29.81918 | 7 | 5.9 | T3b | NEGATIVE |
| GSM18179 | 0 | 69.6 | 7 | 6.8 | T2a | NEGATIVE |
| GSM18179 | 1 | 38.00548 | 7 | 6.9 | T2a | POSITIVE |
| GSM18179 | 0 | 60.72329 | 7 | 35 | T2b | NEGATIVE |
| GSM18179 | 0 | 69.27123 | 7 | 4.6 | T2b | NEGATIVE |
| GSM18179 | 1 | 1.578082 | 7 | 11 | T3b | POSITIVE |
| GSM18179 | 0 | 58.88219 | 7 | 12 | T3a | NEGATIVE |
| GSM18179 | 0 | 60.5589 | 7 | 8.2 | T2b | POSITIVE |
| GSM18179 | 1 | 1.347945 | 7 | 11 | T3a | POSITIVE |
